# Recent Pathological Investigations

**Published:** 1837-07-01

**Authors:** 


### Medico-Chirurgical Review,

No. LIII.

[No. 13 of a Decennial Series.]

APRIL 1, to JULY 1, 1837.

### RECENT PATHOLOGICAL INVESTIGATIONS.

I. Medico-Chirurgical Transactions. Volume the Twentieth. London, 1837.

II. Guy's Hospital Reports. No. IV. April, 1837.

III. The Transactions of the Provincial Medical and Surgical Association. Volume V. 1837.

IV. Outlines of Human Pathology. By *Herbert Mayo*, F.R.S., &c. &c. &c. Parts I. and II.

If it is a difficult, it is a pleasing, and instructive task, to endeavour to diffuse through the medium of the periodical press, that sound and useful knowledge which is accumulating daily. There are some who affect to discover nothing but the progress of sciolism, in the seeming advances of our science. They point to the learning and the laborious study of the men of former days, and reproachfully compare the pigmies of these times, to the giants of those.

Such complaints argue an imperfect acquaintance with the principles that may be drawn from the history of man and of the human mind. In the earlier ages of civilization, the sphere of study is comparatively limited, and one intellect may comprehend all that is precisely known of any or of several sciences. In such ages, so great a deference will be paid to authority, that not to be conversant with what has been written, will be looked on as the worst of ignorance and of presumption; and opinions will be tested, not by their agreement with the facts which men have scarcely learned to value, but by their correspondence with the dogmas which have formed the canons of belief. In that condition of society erudition is deemed the only knowledge, learning almost the sole philosophy.

But when a mind, like that of Bacon's, has burst the scholastic swaddling that first supported, and then cramped the human intellect,—when it has proclaimed that facts are the objects of study, and strict induction knowledge—the necessity for erudition ceases, its utility becomes more dubious—and the philosophic student, turning from authorities that may deceive him, unto one that he believes cannot, diregarding men and worshipping nature, is hurried, perhaps, by a generous confidence in the simplicity of truth, into a culpable disregard for learning.

As facts multiply, one mind is proportionably less able to comprise them, while the growing intelligence renders ignorance painful and probably injurious. Sciolism on the part of the majority is the consequence. But such sciolism is the result of the existence of much knowledge, it is not the evidence of little, and those who cite it as demonstrative of the decline of real learning betray a strange ignorance of its causes and its nature. Lord Bacon was a learned man in *his* day, because he knew most of what was then known, in all the branches of learning and of science. But no human faculties would permit the existence of a Lord Bacon now, of a man who *could* know one-tenth of what is known in those same branches of science and of learning. The age for admirable Crichtons is past, not because the stuff to form them is no more, but because no one man *can* now excel in all things.

Reverting to our immediate subject, we experience no small degree of difficulty in selecting from the works that crowd our table, those which are the best adapted for analysis. It is impossible to make our readers acquainted with all, and we must exercise some degree of discretion, both in giving and withholding. The mass of the profession are too severely occupied, to be able to devote the little leisure they possess to transcendental reveries, and they naturally require at the hands of a Journalist, what will be of practical utility. His aim should be to present in the smallest compass the most important facts.

The works whose titles are placed at the commencement of this article, contain much to interest and to instruct us. In their pages we find the contributions of some of the most distinguished living pathologists, and few of them but are calculated to increase our knowledge on the points which they embrace.

In dignity, perhaps in value, the Transactions of the Royal Medical and Chirurgical Society assume the lead. The present volume is made up of seventeen papers. If we enumerate them, they are :—1, Observations on Vascular Appearances of Mucous and Serous Membranes, as indicative of Inflammation; by Dr. Yellowly. 2, History of a Remarkable Case of Varicose Aneurysm; by Mr. Perry. 3, Case of Recovery from the Insensibility of Intoxication by the performance of Tracheotomy ; by Mr. Sampson. 4, On the Treatment of Injuries received in Dissection ; by Mr. Stafford. 5, Account of a Case of Fracture and Displacement of the Atlas; by Mr. Phillips. 6, Case of Recovery from Poisoning with Opium by Artificial Respiration ; by Mr. Smith. 7, Remarks on two forms of Atrophy of the Heart's Valves; by Dr. Kingston. 8, Case of Unusual Dislocation of the Thigh-bone; by B. Travers, jun. 9, On the Injuries of the Spinal Cord; by Sir Benjamin Brodie. 10, On some Tumours of the Mouth and Jaws; by Mr. Liston. 11, On Ulceration of the Cæcum, &c.; by Dr. Burne. 12, On Black Expectoration; by Dr. Thompson. 13, Conclusion of a Case in which the External Iliac Artery was Tied; by Mr. Norman. 14, Researches on Pulmonary Tubercles; by Dr. Kingston. 15, On some of the Forms of Atrophy of Bone; by Mr. Curling. 16, Sequel of the History of Mary Wren; by Mr. Birch. 17, Removal of a Portion of Lung which protruded through a wound; by Mr. Forde.

The third, the fifth, the ninth, and the thirteenth papers were fully noticed in the last number of this Journal. The remaining papers will be more or less fully analysed in this.

The fourth of the Guy's Hospital Reports, may, without impropriety, be embraced in the same article with the Medico-Chirurgical Transactions. This is no slight honour, and it is creditable to the medical officers of Guy's Hospital, that they should continue to publish regularly such valuable observations as those Reports contain. They have set an example to the Physicians and Surgeons of other Hospitals, which has hitherto met with more praise than imitation.

It is not necessary to allude particularly to the other works upon the list. Their titles, and a portion of their contents are already familiar to our readers, and where we shall make use of them, a specific reference will of course be made to them.

### 1. Affections of the Bones.

The first subjects to which we now beg to direct attention, are the reparation and some of the lesions of bone. The papers which treat of them are:—

1. *An Experimental Inquiry respecting the Process of Reparation after Simple Fracture of Bones. By Mr. Bransby Cooper.**

We need not, we conceive, inform our readers how much attention has at different periods been paid to the mode in which fractured bones are re-united—how many disputes with respect to that mode have arisen—what numerous experiments have been performed—what contradictory conclusions have been drawn from them—nor need we recapitulate the details of the recent observations of Dupuytren, Breschet, and Howship, which have gone far to compose all previous quarrels, silence all exclusive theories, and to bring within the pale of the familiar laws which regulate the nutrition and reparation of the softer tissues, the reparation and nutrition of the bones.

Those observations, though, perhaps, incomplete, have proved to demonstration, or, at all events to the satisfaction of most candid inquirers, that the union of a fractured bone differs little in the manner in which it is effected from the union of divided skin, or muscle: and, that the difference which does exist between them, depends merely on the necessary slowness of an adequate deposition of phosphate of lime.

Mr. Bransby Cooper has been anxious to remove what obscurity still attends the process, and to dispel any difficulties which may yet exist in fully comprehending its details. He has been encouraged to repeat, and, perhaps, improve upon experiments performed by others, by the advice and assistance of Sir Astley Cooper. That excellent surgeon, who possesses in advanced life all the zeal and energy of youth, not satisfied with prosecuting his own useful researches in anatomy and physiology, has put Mr. Cooper in possession of observations formerly made by himself, on the mode

---

* Guy's Hospital Reports, pp. 179, et seq.

of re-union after fractures, and generously allowed him to merge those observations in the present paper. This enhances their interest, if not their value.

"A recent conversation," says Mr. Bransby Cooper, "held with Sir Astley Cooper led me to repeat a series of experiments, which he himself had many years ago projected and performed, and from which the contribution contained in this paper has arisen: and, as no one has thought more deeply, or experimented more extensively, on this subject than Sir Astley Cooper, so did I find that no one was more anxious for its farther investigation, or more sanguine in the expectation of some new light being thrown upon the process by which nature repairs a solution of continuity in the osseous system.

It was at his recommendation, therefore, and with the advantage of suggestions derived from his experience, that I undertook the task of repeating his experiments; the results of which appear to me to possess some novelty, and are certainly such as I had not altogether anticipated. To minds easily satisfied, and to those who think that the present subject has been amply illustrated and explained, it may reasonably be suggested, that even to go over old ground is often profitable, and, perhaps, in the pursuit, something unexpected may present itself." 180.

We should observe that this paper is the first only of a series, and that much may be anticipated from the careful and complete investigation which has been commenced.

Eight experiments have been detailed. Rabbits, as nearly as possible of the same age and size, have been the subjects of them,—the thigh-bone has in all been selected for fracture—this has been done so as to injure the soft parts to the slightest possible degree—and the dissection of each animal has been carefully performed about an hour after its death, in order that, in all, the position of the bone might be similarly influenced by the contraction of the muscles. Five plates accompany the paper, and confirm or illustrate the descriptions.

In the first experiment, the animal was killed 24 hours after the femur had been broken—in the second, 48 hours were suffered to elapse—in the third, the period was 72 hours—in the fourth, it was 96 hours—in the fifth, it was 120 hours—in the sixth, it was 144 hours—in the seventh, it was 168 hours—in the eighth, it was 192 hours.

These experiments only extending to the eighth day after the fracture of the bone, cannot, of course, illustrate the ulterior consequences. But the subject will be pursued by Mr. Cooper, and those ulterior consequences will hereafter be described in a future paper. It is not necessary for us to enter on the details of the experiments before us; an account of the conclusions which may be drawn from them will be sufficient.

1. The first effect of the fracture is extravasation of blood, which fills up the cancelli of the fractured ends of bone, and infiltrates not only the cellular membrane, but even the muscles in the neighbourhood. The blood, by its coagulation immediately produces two desirable results; first, in effecting a degree of stiffness in the injured limb, which prevents the tendency to motion; and, secondly, in restraining a further hæmorrhage, by plugging up the torn blood-vessels, both of the bone and of the softer structures.

2. The coagula begin to solidify, from the absorption of their more fluid parts. The tumefaction of the limb then diminishes. Little change beyond this is to be observed forty-eight hours after the accident, the coagulum only being found firmer than on the examination made at the end of the first four and twenty hours.

3. On the third day, the surrounding tissues begin to be inflamed, from the irritating influence of the coagulated blood; as evinced by the deposition of coagulable lymph, by which the muscles become connected to each other, and their power of contraction diminished, leading to a further means of preventing motion in the fractured ends of the bone. At this period, also, it is to be observed that the coagula, closing the cancelli of the two ends of the bone, are firmly united to the medullary membrane, as well as to the surrounding deposited lymph; and must, in some degree, therefore, assist in maintaining a state of rest.

4. The inflammation induced in the surrounding soft parts still continuing, leads to the effusion of more adhesive matter: at the same time, the ecchymosis becomes less, and paler in colour, and the general surrounding tissues more distinct. From this period, the effused adventitious matter begins sensibly to thicken, and to acquire increased firmness; from a gelatinous effusion, forming a distinct tumor around the fracture, of sufficient solidity to limit still further the motion of the parts, and completely to prevent the muscles being irritated by the irregular ends of the bone. If at this period—namely, between the fourth and sixth days—the callus be examined, it will be found intimately intermixing with the surrounding muscles, at the points opposite to the fractured ends of the bone; and, internally, it is also connected with the periosteum and coagula filling the cancelli, thus isolating the fractured bone from the surrounding structures. After this, all the appearance of ecchymosis entirely ceases; and the only portion of the original coagulated blood which now remains, is that plugging up the ends of the bone, the fibrin of which appears to have become organized; and therefore it may be said, that the original extravasated blood in part assists in the reparation.

5. "About the sixth day, further consolidation ensues; the surrounding muscles appear distinct from the callus, excepting at the points opposite to the ends of the bone. The diffused matter has now both the appearance, firmness, and elasticity of cartilage; and, by its contraction, which occurs simultaneously with its increased density, tends to bring the hitherto separated fractured ends of the bone parallel, although they overlap each other, through the influence of the muscles. At the points where the bones come in contact, the periosteum is absorbed; but in all the other parts, this membrane is inseparably connected with the surrounding mass, much thickened, and easily detached from the bone, so as to give the appearance of the bone having been deprived of its periosteal covering; but the fact is, that it has only become blended with the effused callus. The portions of bone, where denuded, are softened, granular, and seem to be endued with a higher degree of vascularity than healthy bone, probably from the absorption of its earthy constituents. Here it is where the first traces of earthy deposition seem to commence; so that it would appear that the surrounding structures so far assist in the reparation of a fractured bone, as to induce approximation, limit motion, and diminish the irritability and contraction of muscle; while the osseous system itself deposits the earthy matter essential to hardness, the grand characteristic of bone. This view, however, is to be considered as somewhat speculative, as it requires further experiments to prove its validity: at any rate, it is shewn that all the soft parts participate in the injury, and perform a very important office in the completion of a cure:

this is rendered more obvious, by the consideration of the inefficiency of the bones of the cranium, neck of the femur, and some other bones, not surrounded by cellular and muscular systems, to work their own reparation. It is nevertheless certain, that so early as the seventh day, in some instances, and the eight and ninth day in others, earthy matter is secreted in the dense cartilaginous mass in the immediate neighbourhood of the fractured bones. Small specks of the same deposit are observed, at this period, in the fibrin which closes the opening of the broken extremities, and, at a more advanced time, completely shuts up these canals with a firm septum of bone. If sections of the cartilage be made in the mass surrounding the ends of the bone, and placed on glass and dried, the white earthy matter is rendered clearly visible, deposited in minute specks in this more transparent substance." 196.

So far the experiments, and the observations of our author go. At this point both halt, and the resting-place is not a very unnatural one. They have brought us to the commencement of the deposition of the earthy matter, to the process, in fact, of ossification. When the concluding experiments of Mr. Cooper are laid before the public, we shall pursue the subject, and complete our account of the reparation of fractured bone.

In the mean time, we would observe, that this process should be studied, and can only be accurately understood, in connexion with the growth of bone. In spite of all that has been written on the latter subject, much remains for investigation and elucidation, and we believe that patient and ingenious inquiry would be rewarded by satisfactory results.

So far as Mr. Cooper's observations have gone, we are not conscious of their having yet added much to what we know on the mode of reparation of bone. They confirm the opinions now generally entertained, that the surrounding soft tissues become infiltrated with blood, and subsequently with lymph, and that the latter, and perhaps the fibrine of the former, become gradually organized, and the seat of the deposition of the phosphate of lime. The remark, which wanders from received opinions, is admitted by its author to be speculative, and appears to us to be of questionable accuracy. He thinks, that the first traces of earthy deposition are to be met with at the denuded bone itself, which would thus take the lead in the formation of the new bone. But this is contrary not only to what we should expect, from the superior vascularity of the contiguous soft parts, but even to what we have ourselves observed in experiments. We have found the earthy deposition in the fibro-cartilaginous clasp, while the enclosed denuded ends of bone presented no appreciable trace of it.

On the whole, the simplicity of the actual process of reparation of bone presents a striking contrast to the obscurity and difficulties with which the theories and disputes of the earlier anatomists surrounded it; and we may safely say that the only peculiarity in the process, the only circumstances which distinguish it from that of the re-union of the soft tissues when divided, consists in what must *necessarily* result from the slow deposition of the phospate of lime, and from the quantity of solidifying material that is requisite to perform the mechanical office of bone.

II. *Observations on some of the Forms of Atrophy of Bone. By Thomas Blizard Curling, Assistant Surgeon to the London Hospital, &c.**

Mr. Blizard Curling is known to our readers as the author of a work on

* Med. Chir. Transactions.

Tetanus very creditable to his industry, and not derogatory to his judgment. That work was characterised by its exact generalizations on published data, and the present paper is of a very similar description.

Its subject, Atrophy of Bone, is chiefly treated in reference to one or two forms of the affection, which have hitherto been either unnoticed by pathologists, or are only indifferently understood.

The term atrophy implies such changes as are consequent on defective nutrition. But Mr. Curling proposes to employ it here to express all those changes evinced by loss of substance, unaccompanied with any alteration in texture or organization, and without any reference whatever to the morbid action that produces it, since in the majority of cases it is impossible to determine with accuracy, whether the loss is the result of increased absorption or of defective nutrition. And we find it in the osseous system, as in soft structures, under circumstances which seem to indicate that it may be caused in both these ways.

Atrophy, continues Mr. Curling, may occur in the bones generally, or may be limited to certain bones, or to particular parts of bones, and it may be expressed by a deficiency of the earthy particles alone, or by a diminution of all the constituents.

Local atrophy is generally found to be induced by pressure or friction, and is frequently occasioned by the growth of various tumors. The process is probably one of absorption. In atrophy from pressure, the animal and earthy constituents are equally removed. Mr. Curling relates an interesting case, in illustration of this form of atrophy. In the case in question, the head of the right humerus had been fourteen or fifteen times luxated under the pectoral muscle. The bone had been generally replaced without difficulty, indeed without surgical assistance. On the last occasion, in August, 1835, the reduction was very difficult, and, a fortnight afterwards, the patient suddenly died of convulsions. We should observe that though the bone was usually easily reduced, it could not be retained in its natural position. On examining the joint, these circumstances were explained. The head of the humerus was greatly altered in shape, being of an oval form, and its long diameter being in a line with the axis of the bone. About one-fourth of it, together with the connecting cartilage, had been removed so evenly that the head appeared as if a section had been made of it. The inner edge of the glenoid cavity, with its cartilage, was also in great part removed. The portions of bone thus exposed were even and slightly polished. The capsular ligament was smooth internally, apparently enlarged, and loose.

Similar alterations of the head of the humerus have been figured by Sir Astley Cooper, and are preserved in preparations at Bartholomew's Hospital. But all the specimens, four in number, were obtained from subjects in the dissecting-room, and no history of them has been obtained.

"Local atrophy is sometimes the result of an injury, being produced by an action different from that commonly known by the term caries. A highly interesting example of local atrophy of a small portion of the cranium, following a contused wound, has been lately described by Mr. Travers, in the first volume of the St. Thomas's Hospital Reports. Instead, however, of being limited to a part of a bone, or to the seat of injury, atrophy may be occasioned in the entire bone from this cause, so that, without any evident alteration in external configuration, by a change affecting both hard and soft particles, the bone may be rendered smaller and of diminished weight. This, in order to denote the direction of the wasting, and to distinguish it from another form, may be correctly termed *concentric atrophy* of bone. Cheselden, in his Osteographia, has figured the two thigh bones of a soldier shot in the right groin at Gibraltar, who, on returning home the following winter, died soon after of dropsy. The right femur is represented very much wasted as compared with the left, and it is stated to have been less than half the weight of the other." 341.

How local injury operates in producing atrophy is questionable. Commonly the changes to which it gives rise are of a different description. When the functions of the bones are interfered with or suspended, they waste, like the soft parts when similarly situated. This is seen, says Mr. Curling, in the bones of stumps after amputation, and in the bones of anchylosed limbs. In the new Musuem adjoining the Ecole Pratique at Paris, founded by Dupuytren, there is a remarkable skeleton of an adult in which all the bones in the body are anchylosed, excepting the lower jaw and the bones at the shoulder articulations. The bones of the extremities are very much atrophied, the thigh bones being scarcely larger than an ordinary radius.

Bones waste, in common with other tissues, when deprived of nervous influence. A man died in the London Hospital, five months after meeting with a fracture of the twelfth dorsal vertebra, accompanied with such displacement as nearly to obliterate the spinal canal. Sensation and motion were totally lost in the lower limbs, which became atrophied to a remarkable degree, and were so light as to give rise to general remark. Lobstein records the case of a man who died at the age of fifty-four, with extreme atrophy of the right leg, which appeared to be consequent upon a fall received when he was a child, and by which the sciatic and crural nerves were severely injured. On examination, both the soft parts and the bones of the paralysed limb were found reduced to a remarkable state of atrophy. The right thigh bone was found to weigh little more than three ounces, whilst its fellow weighed nearly double; and the gastrocnemius and soleus of the sound limb weighed nearly four times as much as the corresponding muscles on the atrophied limb.

Atrophy is observed to be an effect of a diminution in the proper supply of blood. Such atrophy has been chiefly noticed in the testes, spleen, and kidneys.

The long bones are supplied with blood from two sources—1, from the nutritious or medullary artery, which enters the bone by a distinct foramen, and is distributed to the internal reticular tissue and medullary membrane; 2, from numerous vessels which, after ramifying more or less in the periosteum, are distributed to the outer and more compact tissue.

" Now it occurred to me, that in fracture of these bones one part must have the supply which it derives from the nutritious artery entirely cut off; and although both orders of vessels communicate freely with each other, yet the minute canals, through which the external or periosteal pass, being of a dense unyielding nature, these vessels must be prevented from undergoing that rapid increase in size, which, in the soft structures, constitutes so efficient a provision for a due circulation. I accordingly examined sections of fractured cylindrical bones, in order to ascertain if the ends which had been deprived of their normal supply of blood from the nutritious artery, underwent a corresponding degree of atrophy.

In nearly all the specimens which were first examined, I was agreeably surprised in finding my previous reasoning fully borne out. Thus, in femurs fractured below the entrance of the nutritious artery, I found the interior cavity of the inferior extremity enlarged, the cancelli expanded, and the walls thinned, a form of atrophy which I propose to distinguish by the term *eccentric*. A like alteration was also observed in fractured tibiæ, whilst in a humerus, broken near the middle and somewhat above the entrance of the nutritious artery, the upper portion was the seat of this change." 346.

"This wasting of the osseous tissue in that part of a fractured bone, deprived of its usual supply of blood from the medullary artery is not *constantly* met with, and if the explanation, which attributes it to defective nutrition, be found correct, we should expect that atrophy would not be observed under the following circumstances:—

1. In bones recently fractured: because the process by which atrophy is accomplished must necessarily be gradual, hence some time must elapse before it will be perceptible.

2. In bones long united: because a collateral, if not the regular circulation is subsequently though not immediately established, by which means the previously existing lesion would have been repaired. In old persons, and in those of weak powers, the circulation may never be completely reinstated, in which case the atrophy will be permanent.

3. In bones fractured during the period of growth: because the circulation in bone, as in other structures, being at this time more active, the vessels being larger and more numerous, and the canals, through which they enter the osseous tissue, less dense and but imperfectly formed, the circulation is re-established before any visible degree of atrophy can ensue." 347.

Mr. Curling goes on to say that—

"Another circumstance which must prevent *eccentric* atrophy from being a *constant* result of fracture, is a certain variation to which the nutritious arteries are subject. In some instances, instead of one artery entering the femur towards the superior part of the linea aspera, there is an additional vessel entering below the middle of the bone of larger size even than the superior. There are also occasional variations both in the number, size, and situation of the nutritious vessels of the humerus. In the tibia I have rarely found any variety." 347.

In proof of the preceding statements, Mr. Curling refers to preparations in the musea of St. Thomas's Hospital, Guy's Hospital, St. Bartholomew's Hospital, the London Hospital, the London College, King's College, Mr. Langstaff, and Mr. R. R. Robinson. The preparations in question certainly go to establish the general fact for which Mr. Curling contends, and if there should prove on more extended inquiry to be numerous exceptions, it is something to have pointed out the *tendency* which must still be admitted to exist. So far as the injury has actually gone, it is only fair to state that the exceptions are *not* numerous.

In spite of what has been urged by some surgeons, it is now generally admitted, that the neck of the thigh-bone, when fractured within the capsule, scarcely ever unites by bone—and it is generally believed that one, and perhaps the principal, cause of such non-union, is the inadequate supply of blood to the head and cervix of the bone. The head, under these circumstances, very frequently undergoes a marked diminution in size. The atrophy goes on throughout its whole texture, the cancelli enlarging while the head of the bone wastes. This must be viewed as an instance of atrophy from defective nutrition, and from loss of function, combined with friction.

When atrophy of bone occurs in consequence of the supply from the medullary artery being cut off, no alteration can be perceived in the medulla or its membrane.

There is another kind of atrophy of the neck of the femur, very well ascertained, and very interesting to the surgeon and pathologist. It is the atrophy which occurs in old age, and which commences earliest and goes farthest in this part of the osseous system.

" The atrophy of old age," says Mr. Curling, " is chiefly *eccentric*, the cancelli and medullary cavity first enlarging, and the outer shell subsequently becoming thin, and it is attended with an increased deposition of medulla. In some cases the earthy parts are removed before the animal tissue, which accounts for the head of the femur sinking down upon its shaft and becoming altered in figure. More commonly, however, the texture remaining is brittle, possessing a more abundant share of calcareous matter than at the adult period, so that the external form and size of the bone is preserved, and, instead of yielding to pressure, they break on the application of the slightest force. In either case, however, the bone weighs less than at the adult period.

A change, very similar to that noticed in the head of the femur, as the result of the eccentric atrophy of old age, attended with softening, sometimes takes place in the head of the humerus. But as this bone is attacked at a later period, and is subjected in a much less degree to the influence of pressure, the depression and alteration in figure are by no means so remarkable as in the femur. A good specimen, illustrating this change in the head of the humerus, is contained in the collection of morbid specimens at the London Hospital." 355.

The last species of atrophy of bone which occupies Mr. Curling's attention is mollities ossium. He contributes one fresh case to the existing stock, and has carefully examined and compared those which have been previously published. But we shall not attempt to investigate this curious disease at present, as a remarkable case of it has lately fallen under our own observation, and we propose to lay the details before our professional brethren. We therefore take our leave of Mr. Curling, and proceed to a paper by Mr. Liston, on extirpation of the upper jaw.

III. *Observations on some Tumors of the Mouth and Jaws. By Robert Liston, Esq. Surgeon to the North London Hospital.*

It is very creditable to surgeons and to science, that much less consideration is now attached to operations than was formerly the case,.and that men of great ability and talent for investigation, have applied themselves to what is known in common language as " medical surgery" rather than to the acquisition of mere mechanical dexterity. The latter is, no doubt, an useful and a showy accomplishment, but whoever has witnessed the performance of a sow gelder or the bloody legerdemain of the shambles, must admit that it is possessed in the utmost perfection by the lowest, the most brutal, and most ignorant of men.

We cannot conceive anything more revolting to the mind of a man of intelligence and of humanity, than the reflection that he is regarded as a sort of human butcher, whose hand and not whose head is called into requisition, and who, like an executioner, is only wanted when there is bloody business to be done. Such a man must feel how cheap he is, and deserves to be, held in the world of intelligence and science, and he must own with humiliation that he is little better than a handicraft mechanic.

The history of lithotomy and hernia is sufficient evidence of the extent to which mere operative skill may be carried, amidst the grossest ignorance of all that should regulate the performance of operations. Itinerant rupture doctors formerly made their circuit through the country, practising a mechanical art transmitted from master to apprentice and from father to son, and these bold quacks did not hesitate to operate with confidence and with manual dexterity, for a disease with which they were imperfectly acquainted, on parts, the anatomy of which they did not know.

Surgery, taking its rise from the unwillingness of the educated priest to dabble in blood, and confided at first to menials and to barbers, was long regarded as a mere mechanical art, even when menials and barbers had abandoned it. When we read the works of Mr. Pott, we are struck with the ignorance he displays of medicine, and we constantly observe that if a purgative is to be administered, the physician is solicited to judge of its expediency, and, if expedient, to prescribe it.

To John Hunter is pre-eminently due the praise of having raised surgery to a science and surgeons to the rank of scientific men. No doubt, the general and successful cultivation of anatomy, physiology, and pathology, has tended to the same result, but the relative position of surgeons in this country and abroad, shews how much is to be attributed to the individual exertions and influence of Hunter. In England our best surgeons have a high scientific reputation, quite independently of operative skill, and their consultation-rooms evince how small a proportion the subjects of operations bear to the other patients who consult them. Whatever mechanical merit may be possessed by Astley Cooper, Brodie, Lawrence, Guthrie, Bell, Travers, Green, their reputations are of a higher order, and founded on something better than mere manual dexterity. On the Continent, we see surgery more as it was and less as it should be. The hospital surgeons of Paris are remarkable for their adroitness, and amputate a limb with all imaginable sang froid and celerity, but their opinions before operations and their treatment after it, are notoriously indifferent. The only French surgeon who at all approached the English standard in his *practical* attainments was Dupuytren, and although he fell far short of Cooper or of Brodie in the power of *treating disease*, he was still so much superior to his competitors among his countrymen, that he was regarded as a prodigy. The publication of his Clinical Lectures has convinced the profession in this country, how little of the prodigious there really was in his attainments. Dupuytren, too, was far from a very dexterous operator. He was excelled in many points by Lisfranc, and in some by Roux, yet even in Paris, where a vicious taste, and the imperfection of practical medicine have tended to give a meretricious charm to operations, even there, the sterling qualities of Dupuytren commanded the success which they deserved.

We fear, however, that mechanical dexterity has been undervalued in this country. On the Continent, the performance of operations has been systematised, and every motion, every step has been a matter of investigation and of demonstration. If we have had good operators here, their patent has been like that of—

"God Almighty's Gentlemen,"*

---

* "And as times went then,
His tribe were God Almighty's gentlemen."—*Dryden.*

the produce of human institutions. Yet, surely, the man to whose hands and knife the living flesh of a fellow-creature is to be submitted, should expend some trouble in acquiring that manual facility, which will spare the patient unnecessary pain, and directed and controlled by the higher mental qualities, will conduce materially to his safety. Adroitness is not comparable with those qualities, it is true, but fortunately it may be possessed along with them, and the surgeon who has the higher requisite is inexcusable if he does not obtain the lower.

These remarks have been called forth by the subject to which we are approaching—the tumors of the mouth and jaws.

It is scarcely necessary to remind our readers that the results of the numerous and varied investigations on the nature of the morbid growths and morbid changes of structure may be very briefly expressed. It seems now to be pretty well established :—1, *that*, there are many morbid growths and a few alterations of structure which have no tendency to what is called malignancy ;* 2, *that*, there are growths and alterations of structure, which are decidedly malignant; 3, *that*, there are growths and alterations of structure, which appear to be intermediate between the malignant and the non-malignant: they tend strongly to their own destruction by sloughing or by ulceration, and, so far, they are malignant: but the system shews no disposition to regenerate them when they are removed, or to develop them in other parts, and in that respect they are not malignant; 4, *that*, the physical characters, and the distinctive marks of some of the malignant and non-malignant formations are susceptible of exact appreciation ; 5, *that*, the characters of other new formations cannot, from the examination of their physical structure, be determined with precision ;† 6, *that*, a new formation which has no malignant characters at first, may present malignant characters afterwards: in other words, the non-malignant are convertible into the malignant formations; 7, *that*, the removal of malignant formations by art is, on the whole, extremely unsuccessful and unsatisfactory, for, although, in a few cases, there is no return of the disease, in the majority, it re-appears in some part of the body.

Such we believe to be the conclusions to which well-informed surgeons in this country have come. If they are correct, two important facts are made out:—1, Operations for the removal of the malignant formations are to be undertaken with reserve, and with little expectation of ultimate success; 2, It is difficult, if not impossible, to decide whether many new formations are malignant or are not, and, consequently, if they are subjected to ope-

---

* By "malignancy" is now understood, a disposition in the morbid tissue to be destroyed, sooner or later, by ulceration or by sloughing, *and* a tendency in the system to the formation of similar morbid tissues, whether the primary one is removed or not.

† For example:—we can pronounce that a common fatty tumor is not, and that a well marked encephaloid tubercle is, of a malignant character; but we cannot pronounce, from an inspection of its structure, whether some of the tumors which form in the breast, and many of those which grow from the fibrous or the osseous tissues are malignant or the contrary. We have seen tumors extirpated, which, on examination, had no positive malignant character, and which were looked on as decidedly not malignant, yet true malignant growths have occurred, after the operation, in other parts of the body.

ration, it is equally impossible to predict, with any certainty, the issue. In these latter cases, it is commonly the event, and the event only, which solves the dubious question, If no return of the disease takes place, it is supposed that the disease was not malignant, and, if such a return occurs, it is concluded that it was malignant—an ex post facto, but an unavoidable species of reasoning.

When we reflect on these difficulties which confessedly surround the diagnosis, and the treatment of the new formations, we shall regard with suspicion those formidable operations which have of late years been undertaken for their removal. Such operations exhibit some skill, and more determination on the part of the surgeons who have undertaken them, but it is not so certain that they have always been sanctioned by judgment and deliberation. The details of the operations have been laid before the public, the dexterity of the operator has been the subject of panegyric, but the final results have not, in every instance, seen the light. Before we close these observations we would express our conviction, that if there is a case in which it is necessary to know well *when* to operate, it is that of the morbid growths. A heavy responsilibity lies upon the surgeon, and when the welfare not only of the particular patient before him, but of many other patients, and of science, hangs on his decision, that decision should be founded on other considerations than motives of personal display. We agree, then, with Mr. Liston in the sentiments with which he opens his paper.

"The attention of the profession has been more particularly directed of late years to the diseases of the jaws, in consequence of the novel and bold operations which have been resorted to by some surgeons for their removal and permanent eradication. Many of these affections were formerly looked upon as perfectly irremediable by any means, and indeed I can recall to my recollection several cases which I witnessed when I first embarked in the profession, which were given up as incurable after severe though ineffectual attempts to control their growth by escharotics and actual cautery, which would now be fearlessly and successfully attacked, and effectually removed by operative procedure. Many of the diseases of this region are still, however, in some stages, from their intrinsic nature and disposition, beyond the reach of the science and art of surgery, and one principal object which I have in view in the following paper is to point out what I consider to be the characters by which those tumours which may with safety and propriety be interfered with, may be discriminated from those affections, on the other hand, which no conscientious or well disposed surgeon ought or would think of touching with a knife. I hold it to be a maxim never to be forgotten or departed from, that no operation, far less one hazardous to life, should be entered upon, unless there be a fair prospect and strong probability of ultimate success. No man is entitled to put the life of another in jeopardy unless, after all the suffering and risk attendant upon this last resource, success is likely to crown his efforts.—In all diseases, wherever situated, of a malignant tendency, when from their extent and duration there is a certainty, or even a probability of the neighbouring parts being affected, or so disposed as to take on the same action, or where the lympathic system is contaminated, very little prospect or hope of successful issue can be held out. We are therefore called upon to look to the previous history of such diseases, their origin, progress, and duration,—to the signs of the malady,—and from these to form, so far as we are able, a correct diagnosis." 166.

We now proceed to give an account of Mr. Liston's observations on the tumors of the mouth and jaws, observations intended to elucidate their nature, and to discriminate the malignant from the non-malignant forms.

1. He alludes to parulis and to spina ventosa. The latter name is almost obsolete, the thing described by it uncertain, the pathology of it unsatisfactory, and the treatment, consequently, dubious. On this head Mr. Liston says nothing new, nor does he make any additions to our knowledge of epulis.

2. But, he says, " A disease of a more troublesome nature seems however to proceed originally from the sockets of the teeth, from the periosteum of the root. It appears as a soft vascular growth from the apex or side of the fang, (the soft medullary fungus filling up and projecting from the hollow of a decayed tooth seems to have a different origin) : this is loosened, the gums separate and become injected and flabby, they swell out and form a tumour investing the teeth, which one after another become displaced, and after being removed, their roots are sometimes found highly injected with blood. Copious sanious discharge is furnished from the soft and fungous mass, which bleeds on the slightest touch; the osseous tissue, comprising the alveolar processes, and even the substance of the jaws, is changed into a soft lardaceous or brain-like mass. Of this disease in its incipient and advanced stage, there are to be seen specimens in many collections. It is not improbable that many of the more solid and less malignant diseases, involving the upper and lower maxillary bones, have a somewhat similar origin.

The progress of these diseases is various; in some it is rapid, and the growth is soft and unlimited. It soon bursts through the walls and investments of the bone, adhesions are formed to the neighbouring parts, which take on an unhealthy action, a fungous growth of a soft and bad character is thrust out into the mouth, the lymphatics are soon affected, and the case is speedily in a hopeless condition." 169.

The preceding observations corroborate, so far as they go, what we have already urged, that the diagnosis of the malignant and non-malignant tumors, more especially of those which spring from the fibrous structures or from bone, is involved in difficulty and obscurity. Mr. Liston first of all admits, that the tumors which go by the name of epulis, though not generally " mali moris," " occasionally do degenerate, contaminate the neighbouring parts, and are liable to be reproduced, and sometimes after some interval from their apparently complete extirpation." In other words, epulis is sometimes malignant and sometimes it is not, and no characters are mentioned by which we may decide the question.

In the second place, Mr. Liston, after describing a malignant growth, allows that it is not improbable that "many of the more solid and less malignant diseases, involving the upper and lower maxillary bones, have a somewhat similar origin." Thus, physical structure is no certain criterion, for epulis sometimes turns out to be malignant, and mode of origin is no certain criterion neither, for malignant and non-malignant growths may have a similar one.

3. " In the upper jaw," continues Mr. L., "a most malignant and intractable disease is met with, often traceable to long continued irritation in the alveolar processes or sockets, from the presence of decayed or bad portions of teeth. The disease, it is probable, sometimes commences in the manner already indicated, and spreads to the lining membrane of the maxillary antrum, or it may and must often have its origin in that tissue originally. We find, in fact, though more rarely, the same morbid structure springing out of the frontal and sphenoidal sinuses primarily. This disease is so well known that it would be out of place here to enter into a description of its progress or anatomical characters. Its accession is insidious, the patient has painful sensations in the side of the face, a feeling of distension, there is some increased discharge from the nostril, and the cavity becomes obstructed. Lodged deeply amongst the bones of the face, it makes its way at first very gradually, drawing into the same action the investing parts, the bone and its exterior covering, the teeth become loosened, the walls of the cavity are protruded and softened, œdematous matting and discoloration of the cheek follows, fungous growths shoot out into the nose, towards the mouth through the thin anterior parietes, or through the palate, presenting a soft, ragged, foul ulcer, (not very appropriately designated cancerous by some writers,) through the tuberous process backwards to the throat, and ultimately towards the eye through the floor of the orbit, extruding this organ, and involving it in a frightful, soft, and sometimes bleeding mass. To this disease the term osteo-sarcoma (not a very good or appropriate one in any instance) has been often applied. The bone is involved secondarily in many cases in the morbid mass, is expanded, softened, and wasted by its blasting influence. It is not a tumour of bone or a conversion of it into flesh, or pulpy matter. The march of this disease is usually exceedingly rapid. Some tumours in this situation, soft and brain-like, and from the first of a bad kind, grow more slowly and have no great tendency to bleed." 170.

This is a description of undoubted malignant disease. Yet even in this there are varieties, for sometimes its progress is tardy, sometimes rapid—sometimes it is soft, and sometimes comparatively hard—sometimes it springs from the alveoli, sometimes from the sinuses—sometimes it originates in one tissue, sometimes in another.

4. Tumors of erectile tissue have been found to occupy the antrum. They are rare.*

" But the superior maxilla is found now and then to be involved in a tumour of a more simple and manageable nature, commencing in the osseous structure or periosteum. Some rare cases also of extensive deposit, of very hard osseous matter ultimately filling up the cavities and fossæ of these bones, are occasionally met with. The fibrous or fibrinous tumours, as they are more properly denominated by that indefatigable pathologist Mr. Kiernan, are very generally traceable to some external injury, (to which indeed the majority of enlargements of parts and new growths in all situations are to be attributed,) and are of comparatively slow growth. From its more exposed situation, the lower jaw is more frequently found affected with swellings of a simple or fibrous character than the upper, and it may be deduced from what has already been remarked, that the affections of the latter bone are generally of a malignant kind ; those of the former more benign. As it has been already remarked, however, simple tumours involving the upper jaw and neighbouring bones, principally the os malæ are occasionally met with, and the lower jaw is not exempt from the attacks of the softer and more troublesome degenerations.

The simple tumour, whether involving the upper or lower jaw, differs in consistence and in form also from those soft, lardaceous or pulpy and brain-like masses, whose appearance and progress I have shortly alluded to. They attain though slowly a great size, they present a *globular* or *botryoidal* form, displace

---

* One of the most remarkable is that related in Pattison's edition of Burns' Surgical Anatomy of the Head and Neck.—Rev.

the surrounding soft and hard parts, project from the countenance, and deranging the features produce great and frightful deformity. The skin may be thinned and pervaded by enlarged venous branches; it is discoloured, but not incorporated even in an advanced stage with the morbid mass, nor are any of the surrounding tissues contaminated. The projection towards the mouth, often large and passing down by the side of the opposed teeth and jaw, is hard and elastic, and conveys the feeling of brawn interspersed with bony particles; but it is covered by a continuation of the mucous lining of the cavity slightly thickened and altered, furnishing an inconsiderable discharge, and that neither offensive nor of a bad quality. This growth in the mouth presents indentations made by the teeth with which it comes in contact. The hard palate when the upper jaw is involved, is generally covered by a thick layer of tumour, which projects over, and lies in contact with, but is not adherent to it, nor to the gums supporting the teeth of the opposite side. It obscures the view of the velum and fauces, and by impeding respiration makes the patient very uncomfortable, renders his supply of nourishment incomplete, and even puts his life in jeopardy. The tumour of the lower jaw again, by the displacement of the tongue and interruption to the performance of its functions, is equally inconvenient and dangerous. In the records of surgery, I can find very few such tumours described as affecting the superior maxilla, and my enquiries respecting the cases which have been subjected to operation, which are as yet unpublished, would lead me to conclude that the diseases interfered with, have not all been of this benign and tractable nature. In many of these the morbid action has not ceased, the growth has been reproduced, and the patients have not in any way been benefited by the treatment." 173.

Such is Mr. Liston's description of the non-malignant tumor of the upper jaw. He very properly repudiates as a general proposition the attempt to remove the malignant tumors by the knife. He stigmatises it, and not too coarsely, as fool hardiness. It is worse. Fool-hardiness is a term which is applicable to one who does a foolish act which may be injurious to himself. But when that folly is at another's expense, the vocabulary must be searched for some more damning epithet.

But as Mr. Liston limits the propriety of operating to tumors which are not malignant, and as he attempts to describe their characters, it is necessary, of course, to examine that description, in order to ascertain if it be sufficiently precise to prevent the risk of misconception.

The distinctive features of the simple tumor appear, on analysis, to be the following:—1, its slow growth; 2, its great size, and globular form; 3, its hardness; 4, its fibrous, or fibrinous structure; 5, its indisposition to convert to its own nature the tissues with which its growth brings it into contact.

It must be owned, that the *ensemble* of these characters is that of a non-malignant tumor, and that wherever they characterised a growth, that would be considered, cæteris paribus, not very ill adapted for an operation. But while this general admission must be made, it does not follow that the distinctions between this and the true malignant tumors are too marked to prevent the possibility of error. Experience has proved, with respect to tumors in other situations, that neither slow growth, a fibrous structure, nor indisposition to involve contiguous parts, are *certain* guarantees of the absence of malignity, and he must be a bold pathologist, we might almost add an ignorant one, who would say of such a growth as has been described by Mr. Liston, that it would not and could not contaminate the constitution, or be succeeded by similar growths when removed.

Practically, however, the question is not a broad one. If it is granted, and we think it must be so, that the characters of the tumor are, on the whole, not such as would preclude an operation, it is the surgeon's duty, supposing an operation practicable, to perform it at as early a stage, and under as favourable circumstances, as he can.

Mr. Lizars first shewed that an operation for the removal of the upper jaw is practicable, and he pointed out the method of performing it.

Mr. Liston has ascertained that fifteen cases have been laid before the profession and the public, in which it appears that the whole or greater part of the superior maxillary bone has been exirpated, (in one of them, by the way, incisions were made for this purpose on two separate occasions, and but little of the disease removed). Eleven of these patients died, either from the immediate effects of the operation or from a return of the disease. The operators were Messrs. Lizars, Gensoul, Syme, Robert, Scott, Earle, Guthrie, and Hetting. In two of the four who are said to have been cured, the tumor is admitted to have been soft, and probably of a bad kind. In fact, out of the fifteen, one case only appears to have been, at the period when interfered with, very favourable for the operation; the first, viz., recorded by Gensoul. That tumor he describes as globular, of firm consistence, fibro-cartilaginous, and of a homogeneous structure. M. Gensoul's second case also seems to have terminated favourably, and is one of those already alluded to as composed of erectile tissue.

Different modes of operating have been adopted, and Mr. Liston offers the following short account of them.

"The tumours of the gums cannot be satisfactorily or permanently removed unless the teeth which they embrace are extracted, whether these be in a sound state or not. Indeed some slight and more simple swellings will subside on the removal of the remains of diseased teeth, which have acted as the exciting causes of mischief. There can be no doubt as to the propriety and absolute necessity of taking away all source of irritation as a preliminary step to operations of any kind in this cavity, whether the disease has arisen in the mucous surface, in the substance of the lips or tongue, in the glands, gums, or osseous structure, or in the cavities contained therein.

An incision should be made with a strong pointed knife, so as to surround the base of the tumour, and wide of the morbid structure; when the alveolar processes are involved, these must be cut away with cross cutting forceps, and if to any depth, perpendicular sections should be previously made on each side of the mass with a fine saw. When there is reason to dread that the structure is at all of a bad kind, when unsuccessful operations have been previously practised, besides the free excision, it will be advisable, after the oozing of blood has ceased, to apply either the actual or some potential cautery to the exposed surface. In many cases this is not demanded, as a permanent cure is often found to follow the clean extirpation. The incisions either in the upper or lower jaw, must necessarily be made so free as to take in the whole morbid mass. I have found it necessary to remove extensively the floor of the antrum and to cut down the alveolar ridge of the lower jaw, leaving merely a thin line of the base, in order to attain this end in some cases. This proceeding is preferable, when it can be accomplished to the cutting across and removing the whole thickness of the bone; deformity is thus in a great degree guarded against, and the corresponding teeth of the upper and lower jaw meet. But even after the removal of great portions of this bone, as by disarticulation, and by some attention on the part of the patient, (he being provided with, and being directed to wear for some time and during the night, caps fitted to the teeth of the sound side above and below, and soldered together,) the remaining half of the bone, during the cicatrization within the mouth, is prevented from being drawn awry, and much annoyance is thus averted.

The malignant tumours, those of soft consistenee, which lose themselves insensibly in the neighbouring tissues, if interfered with at all must be attacked by some very effectual operative procedure, and in their very earliest stage. The fore part of the antrum so affected, as already noticed, has been exposed and opened in very many cases by incisions of the cheek and the morbid growth been taken out, and this has been followed up by the application of strong escharotics or of cauteries. The result of such operations has seldom been at all satisfactory, as can be readily understood. The difficulty, even in favourable cases, of taking away after this fashion the whole of the contaminated parts is very great, and there is reason to fear that very frequently the progress and fatal termination has been, under such circumstances, accelerated." 177.

Mr. Liston relates four cases, and glances at two others, in which the superior maxillary bone was removed on account of a morbid growth. It is not necessary for us to enter on the details of these cases, and we shall merely extract the particulars of the operation in one, in order to shew the steps pursued in the removal of the tumor.

*Case.* It is necessary to mention the form and the dimensions of the tumor, in order that the course of the incisions may be more readily apprehended.

The tumor is as large as a moderate-sized cocoa-nut, causing great deformity. The mouth is drawn to one side, and the vision of the left eye partly obstructed. She complains of very little pain, and her general health is good. The swelling is of firm consistence, and appears to involve the whole of the left superior maxillary bone; internally it occupies the whole of the palate, but is unattached on the right side; a probe can be passed under it for some distance. It extends as far back as the finger can reach, and projects over the velum, concealing a great part of it.

The operation was performed as follows :—

" After removing the central incisor of the left or opposite side, which was rendered necessary by the extent of the tumour, Mr. Liston commenced an incision a little below the inner angle of the eye and carried it obliquely under the corresponding ala of the nose, detaching its cartilage from the bone, then through the lip into the mouth, in the mesial line. An incision was next made from the prominence of the cheek to the angle of the mouth. The flap thus formed was reflected upwards. The tumour, it was now ascertained, extended considerably backwards, and it was necessary to make another incision, nearly at right angles, on the outer perpendicular one, in the line of the zygomatic arch: the tumour was thus exposed throughout its whole extent. With strong cutting forceps the nasal process of the superior maxilla was divided; the operator next cut through the zygomatic arch, near the auricle, and then through the malar bone at the transverse facial suture into the spheno-maxillary fissure; the diseased maxilla was separated with great facility from its fellow of the opposite side, by strong scissors, leaving the palatine plate of the palate bone and velum palati entire and untouched; the superior maxillary nerve was carefully divided. The diseased mass was now readily removed, involving the whole of the superior maxillary bone, the whole of the malar inferior spongy bone, and the zygomatic process of the temporal bone. The internal maxillary artery, which bled not very freely, was immediately tied, and the edges of the flap brought together by five points of twisted and two of interrupted suture." 191.

II. On Inflammation, Chronic Disease, and Perforative Ulceration of the Cæcum, and of the Appendix Cæci, with Symptomatic Peritonitis and Fæcal Abscess. By *John Burne,* M.D. Physician to the Westminster Hospital.

The fault of the present day is certainly not "abandonnement de soi," nor are pathologists more remarkable for that weakness than their neighbours. Every one, in the medical world, is anxious to be esteemed, is willing to believe himself, a discoverer. We see this anxiety manifested in every conceivable way. Your hospital surgeon affects an entire ignorance of all that is done or that has been done by others, and delivers a clinical lecture, or publishes some clinical reports, in which he dwells with most becoming importance and solemnity on results of *his* experience, which, oddly enough, every reader, not actually a neophyte, discovers, to have been the results of *his* experience too. The physician, if of a practical turn, has hit on some nice points of diagnosis, which may, possibly, have been hit upon before, but which he verily *believes* never were so. The pathologist, the knight-errant of medicine, "his eye in a fine phrenzy rolling," has found in some tissue a little more white or a little more black, than he *conceives* other pathologists have; and is he not a discoverer? Nay, the merest routine practitioner has the genius of invention too, for he has a mixture or a pill, which suits the debilitated old ladies or the hysterical young ones, far better than any former, or any contemporary, pill or mixture. Look which way we will, we see novelty stare us in the face, and it is gratifying to think not only that there is so much of it, but that there is so much genius afloat.

Yet the cynic is disposed to observe, that the industry displayed in collecting and in publishing *original* facts, is very disproportioned to that exhibited in ascertaining and recording the previous researches of others. An author has two eyes of very different refracting powers. One is microscopic, and with that he deciphers his own page, in which the minutest letter acquires size and importance. The other has the faculty of diminishing all great objects, and of rendering ordinary ones imperceptible, and with this eye he reads the pages of his rivals. Authors may claim the privilege of forming a part of the providential scheme, which provides for the continuance of what is valuable on earth. They resemble those birds which swallow grain, and voiding it at a remote period and in a distant place, in the shape of excrement, are the useful means of disseminating the seed.

But we must turn to the paper before us. Dr. Burne is an intelligent and a zealous physician. He has cultivated pathology with assiduity, and has, at different times, communicated the fruits of his labours to the public. His reason for writing on the present subject is briefly stated in the following paragraph.

"The diseases of the cæcum and of the appendix vermiformis, which form the subject of this paper, have received no separate consideration in the systematic works on practical medicine; nor have I met with any notice of them except in the medical periodicals, in which detached cases have from time to time, been published." 200.

Abscess in the iliac fossa connected with or dependent on ulceration of the cæcum has been made the subject of more elaborate notice, than the preceding extract would lead its readers to suppose. Four distinct articles upon the subject have appeared at various times in this Journal, a memoir on it has been published by M. Tealier in the Transactions of the Societé Medicale of Paris, and M. Dupuytren has expressly treated of it in a distinct chapter in his Leçons Orales.* But, perhaps, Dr. Burne has drawn more attention than had previously been done to inflammation of the cæcum, unaccompanied by perforation or by abscess in the contiguous cellular membrane. We shall, therefore, notice this portion of his paper, and pass over in a cursory manner the more familiar division of it.

A. "An idiopathic inflammation of the cæcum," says he, "from the ordinary general cause, exposure to the vicissitudes of the weather, has not fallen under my notice. The inflammation of which I am about to speak has in every case been symptomatic of some mechanical exciting cause, as the lodgment of undigested food, of fruit-stones, or of concretions, which the structure of cæcum and appendix favours; and hence the peculiar features of the disease." 202.

The operation of foreign substances in determining irritation, and, consequently, inflammation of the cæcum has been strongly insisted on by M. Dupuytren, who was, perhaps, the first to draw attention very forcibly to these affections.

"The affection," says that author, "is infinitely more common on the right side than on the left, which is evidently due to the anatomical relations of the parts. On the right side, the cæcum is comparatively little invested by the peritoneum, its posterior part is invested by a loose cellular membrane, and where it joins the small intestine (at the valve of the colon) a sudden contraction of the tube takes place, forming a kind of nest for the lodgement of irritating foreign bodies, as plumb-stones, cherry-stones, &c."

To revert to Dr. Burne, and to the case:—

"In all the examples of inflammation of the cæcum which I have witnessed, the development of the symptoms has been in the following order. The first sign is a sense of uneasiness which soon amounts to an aching pain, deep-seated in the right ilio-inguinal region, arising unexpectedly while the person was in health, and not preceded by rigor or exposure. This pain increases progressively for twelve or twenty-four hours, retains its character, is fixed and constant, never even remitting. Then supervene gradually tenderness, fulness, and tension of the whole ilio-inguinal region; the bowels are constipated and do not reply to medicine, and the patient grows sick and vomits. Some febrile movement now begins to manifest itself, the tongue becomes white and furred, the urine scanty: the appetite is gone, the pulse is frequent tight and sharp with increased volume, but the stroke though sharp is not strong, nor is its impression on the finger decided, it is a pulse of irritation and inflammation combined; the patient lies on the back quite still, slightly inclined to the side affected, and the case presents a serious aspect.

This state of things will persist for several days, the pain remaining of a very severe aching character. The fulness and tension of the part will increase and extend to the other regions of the abdomen, which hitherto had been soft and

* We have ourselves reported several cases of the kind, and made, at different times, some observations on the subject.—*Eds.*

not tender; and a sign will now be present in an eminent degree characteristic of this inflammation, it is an *exquisite tenderness* of the abdominal parietes covering the cæcum, a tenderness far exceeding that of an enteritis, even of a peritonitis; the patient will scarcely allow a finger to be laid upon the part; when you go to examine it he lays hold of your hand and supplicates you in an earnest manner not to touch it, not to hurt him; he cannot bear the weight of the bed-clothes upon it: the tenderness is as exquisite as in any case of acute rheumatism I ever saw. The constipation continues, but the vomiting does not become frequent and distressing as in enteritis, nor does the face betray so soon the anxious aspect. Taking the case altogether, it is not such an imminent affair of life and death as an enteritis, though in the sequel it may prove equally fatal. Such are the signs of the symptomatic inflammation of the cæcum." 204.

Dr. Burne's remarks are more precise, as we shall shew, than M. Dupuytren's, who thus enumerates the premonitory symptoms of suppuration in the iliac fossa.

"L'apparition de ces tumeurs est souvent précédée de symptômes précurseurs qui annoncent le développement prochain de la maladie. A la suite de quelques erreurs de régime, d'une constipation ou d'une diarrhée plus ou moins prolongée, de coliques plus ou moins habituelles, quelquefois sans qu'aucune de ces causes ait paru, la malade éprouve des coliques plus violentes et des douleurs d'entrailles qui ont une tendance à se concentrer dans la fosse iliaque droite; elles peuvent aussi 'irradier dans la direction du gros intestin; ou bien être disséminées dans toute l'ètendue de l'abdomen.—Ordinairement ces coliques sont accompagnées de constipation, et dans quelques cas, de vomissemens.—Tels sont les signes á l'aide desquels on peut prévoir l'apparition de la tumeur. Leur durée varie beaucoup, et l'on voit des malades qui en sont tourmentés pendant six semaines, deux mois et plus, tandis que d'autres ne les éprouvent que quelques jours avant l'invasion de la phlegmasie." 334.

If alvine evacuations can be procured, and the symptoms subside, which seldom occurs in less than seven or eight days, the termination of course is favourable. Occasionally evacuations are not obtained, the symptoms do not subside, and the patient sinks rather from exhaustion than from inflammation; or, ulcerative perforation of the cæcum occurs, and a circumscribed emphysematous tumor presents, either in front, in the right ilio-inguinal region, or posteriorly in the right ilio-lumbar region; this tumor being a fæcal abscess which is making its way to the surface of the body. If the perforation has taken place on the anterior part of the cæcum, adhesion will have formed around the perforations, and thus the fæcal abscess will arrive at the circumference of the body without involving the peritoneum in a general inflammation; if of the posterior part, which has no peritoneal tunic, then the peritoneum will escape altogether, and the abscess tend upwards and backwards to the least resisting part of the lumbar parietes, which is at the outer edge of the quadratus lumborum muscle. This abscess may discharge itself and the patient do well; or nature may be unequal to the task, and the patient sink exhausted.

"The *diagnosis* of these cæcum cases is determined with precision by the seat of pain, the exquisite tenderness, and the tension—by the sudden invasion of the symptoms while the person was in health—by the local signs preceding the development of the febrile movement—by the degree of fever being less than in idiopathic inflammation, and by the less marked anxiety of the countenance.

If any doubt remain, it will be dissipated by laying the hand upon the part, when the circumscribed fulness and hardness in the region of the cæcum will give assurance of the seat and nature of the affection. A practitioner who witnesses one of these cases for the first time is satisfied it is not a common inflammation of the bowels, although he does not know its exact nature: he says the case is a curious one; he cannot make it out." 205.

The treatment may be readily conceived. As the inflammation is the result of a mechanical source of irritation, or, perhaps, obstruction, it is obvious that depletion must not be carried to so great an extent as in idiopathic enteritis. Another consideration which should moderate the employment of depletory measures, especially of local or general blood-letting, is the reflection that the patient may have to go through an iliac abscess, and that his powers should be husbanded for its support. The depletion then should be cautious—enemata, and such purgatives as the stomach will bear well should be administered*—light poultices and fomentations are to be applied—and about the fifth or sixth day the bowels may begin to act, and the symptoms to subside.

Should they not subside, the physician or surgeon must anxiously watch for the first appearance of an emphysematous tumor, and make an early incision into it.† Fetid gas escapes, and the cellular tissue is more or less sloughy, or actually sloughing,‡ The patient must now, of course, be supported, and even stimulated, to the necessary pitch.

Two cases are related, but they need not detain us, and we proceed.

B. "It is a fact," says our author, "established by pathological anatomy, that those parts of the intestinal canal where its dimensions vary and its organization changes, are particularly liable to be affected with chronic disease. The cæcum is one of these parts; and in addition to the acute inflammation which has been described, it is not unfrequently the seat of a subacute chronic inflammation or pathological congestion, which induces thickening of its tissues and contraction of its natural capacity, impairing thus the organization and function of the gut, so as to render the action of the bowels irregular and difficult, and eventually to determine a complete and fatal obstruction. This diseased condition of the cæcum may exist for several months or even years, accompanied always with alvine difficulty and sympathetic disturbance of the stomach, of the same character exactly as that which attends stricture of any part of the alimentary canal. The sufferings are similar, the health declines, the body wastes away, and patients die worn down by sickness, inanition, and the dreadful spasmodic pains arising from the violent efforts of the bowel to overcome the obstruction. When emaciation has arrived at a considerable degree, the powerful peristaltic action may be felt, even seen, heaving the abdominal parietes in tracts corresponding with the convolutions of the intestines." 212.

A case is detailed in illustration. The patient was received into Guy's

---

* Dr. Burne considers one drachm of the sulphate of soda in half an ounce of the infusion of senna, with four drops of the tincture of opium, a good form. In case milder remedies fail, he recommends extract of colocynth six grains, calomel two grains, opium one grain, to be exhibited every six hours.

† Singular as it may appear, it has been a matter of dispute whether the incision should be made early or late. Analogy, every thing is in favour of its being early.

‡ We have in two instances seen artificial anus in the groin ensue.

Hospital, and died there. On dissection, the cæcum was found to be thickened and much contracted, the colon was empty and contracted too, while the ileum was distended with feculent matter and flatus. It is curious that, in this case, the sigmoid flexure of the colon stretched across the abdomen to the right side, and was united by recent adhesions to the cæcum and the ileum.

In another case, the cæcum was contracted and thickened, its tunics being blended together, and transformed into a dense, opaque, white, unyielding gristly substance; and interiorly were discovered numerous organized bands covered with a smooth shining membrane, stretching across the channel of the gut from side to side, in various directions, forming an irregular coarse net-work. In this contracted cæcum and net-work, a complete obstruction had been formed by fæculent matter, dry and friable, plugging up the channel in a manner which nothing could have removed. Leading to this part was the ileum, filled with an amazing quantity of soft, yellow, homogeneous fæculent matter, the bowel itself having all its tunics confounded together, and converted into a dense strong tissue, a line in thickness, resembling thick wet parchment: all trace of villous structure, or valvulæ conniventes, having disappeared. In addition, we should observe that the convolutions of the intestines were agglutinated together by soft lymph.

We need not mention the particulars of a case of fæcal abscess, consequent on ulcerative perforation of the cæcum.*

c. The appendix cæci vermiformis is, like the cæcum, liable to be diseased. "The conformation and situation of the Appendix," continues Dr. Burne, "vary much in different individuals, a fact not noticed by anatomists, but which I have found to influence the phenomena and nature of its diseases very considerably. The conformation of the appendix is generally described as flexuous, and its situation as depending into the pelvis, but by some the situation is not noticed, further than that the appendix arises from the cæcum and is bound down to it on the right by a fold of peritoneum, the meso-appendix; whereas the appendix is more frequently situated on the outer edge of the psoas magnus on the facia iliaca, snugly curled up beneath the cæcum and concealed by it; a fact which I have verified by many dissections, and one of great importance to the pathologist, as will be seen. In the event of a perforative ulceration of the appendix, and a consequent peritonitis or fæcal abscess, the parts involved will differ entirely according to the situation of the appendix. If it should happen to depend into the pelvis, then the pelvic viscera will be implicated; if it should happen to be situated on the iliac fascia and underneath the cæcum, then the belly of the iliacus internus and the neighbouring adipose cellular tissue will be involved, and the course of the abscess be determined accordingly: so important is the relative anatomy of even inconsiderable organs to the physician. In a case to be related where the abscess pointed in the loins, the whole belly of the iliacus internus was in a state of gangrene, as was also the mass of adipose

---

* Mr. Henry James Johnson has published a curious and interesting case in which the following circumstances occurred. Thickening and ulceration of the cæcum, ended in perforation of it. Its contents partially escaped into the iliac fossa. Then the pus gradually found its way by a sinuous passage in the cellular tissue between the transversalis muscle and the peritoneum to the bladder—a small ulcerated aperture occurred in its anterior wall—a little pus escaping into its cavity rendered the urine albuminous—excessive irritability of the bladder was established—and, apparently worn out by this, the patient died.

tissue in the lumbar region: the abscess was here making its way to the outer margin of the quadratus lumborum muscle, the part where the parietes offer least resistance to its progress." 220.

Foreign substances may pass from the cæcum to the appendix, and lodge there. If small they may excite no irritation at all, or ulceration of the mucous membrane only. But if, from their size, or from any other cause, the peritoneal coat is involved, it is obvious that formidable or fatal peritonitis may follow, or, if the surrounding inflammation is circumscribed, a limited abscess may ensue. This may come forward, burst, and discharge itself—or it may remain stationary, and occasion serious or fatal irritation of the system—or, it may burst into the cæcum.

Dr. Burne details three cases which will repay the perusal of those who will consult them in the original paper. The paper itself is an useful one. It systematises our knowledge of the primary affections of the cæcum, and of their secondary consequences, and we have given a full account of it because we deem it valuable. As we previously observed, Dr. Burne is a diligent pathologist, and an observant physician.

### III. AFFECTIONS OF THE HEART AND ARTERIES.

1. *Remarkable Case of Varicose Aneurysm. By John George Perry, Surgeon to the Foundling Hospital, and to the St. Marylebone Infirmary.**

By varicose aneurysm is meant a communication by means of an intervening aneurysmal sac, between an arterial and venous trunk, the blood of the former passing through the sac into the latter. The communication in question has hitherto been regarded as necessarily the result of a wound, there being no recorded case in which it took place as a consequence of ulceration. But the following *is* such a case.

*Case.* J. Allum, æt. 47, had been discharged from the Dragoons, in 1819, on account of ill health. Prior to this, he had been knocked down and his left knee had been hurt. In 1831, being at that time employed in drawing a truck, he suffered for several months from pain in the foot, at its inner side, and matter formed, and was discharged from that part. Sometime in the course of the same year he first perceived a small swelling a little below his left knee, but it gave him no pain and he disregarded it. The swelling increased very slowly in size and troubled him very little, that he could recollect, for two or three years, except that occasionally, when drawing his truck, he was seized with pain in it to such a degree as to be obliged to stop for a few minutes from his work. In 1833, his wife accidentally perceived a "palpitation" in the middle of his left thigh; he was subject at the same time to palpitation of the heart. The former increased, the inner side of the foot and great toe became again extremely painful, and on the 6th of February, 1834, the patient was admitted into the Marylebone Infirmary.

"Upon examination soon after his admission, an aneurismal tumour of con-

* Med. Chir. Transactions.

siderable size was discovered at the upper part and inner side of the calf of the left leg, apparently occupying the lower end of the popliteal, or the commencement of the posterior tibial artery. The contents of the sac were so entirely fluid, that it could be almost emptied by pressure with the hand, maintained for a few minutes. Pulsation could be felt distinctly in the anterior tibial artery as it passed over the tarsus, and obscurely in the posterior tibial, behind the internal malleolus.

As the patient lay on his bed with the limb placed on its outer side, a very remarkable pulsation could be distinctly *seen* along the course of the femoral artery and vein throughout the greater part of the upper two-thirds of the thigh, beginning about two inches below the crural arch, and terminating at the spot where the femoral vessels become included in the tendinous sheath formed by the triceps muscle. When the hand was laid upon any part of this region a very peculiar thrilling was perceived, occupying a space of at least two inches on either side of the vessels, but varying in intensity and force according to the distance of the part examined from them. I know not how to describe the sensation communicated to the hand better than by borrowing a simile from Laennec, who, in describing the sounds attendant on a contracted state of the left auriculo-ventricular opening, says, 'on sent quelquefois à la main un frémissement analogue à celui qui accompagne le murmure de satisfaction que font entendre les chats, lorsqu'on leur passe la main sur le dos.' This purring (to adopt his phrase) was quite distinguishable from the pulsation of the artery; for while the latter was lost in the intervals corresponding to the diastole of the heart, the former continued to be felt without intermission, deriving only renewed intensity from the repetition of the heart's action." 35.

Much difference of opinion existed, on the cause of the phenomenon. In one of the examinations made by Mr. Perry, he accidentally discovered that pressure with the finger upon a part of the artery, immediately before it entered the sheath of the triceps, immediately stopped the purring, without interrupting the circulation through the vessel. This suggested the idea of a communication between the artery and vein, but the patient denied the previous occurrence of any wound that could occasion one.

The diseased state of the vessels precluded, in the opinion of all, an operation. Pressure, in various ways, was accordingly tried, but occasioned too much uneasiness to be borne. After remaining nine months in the infirmary, the patient quitted it. On the 22d of July, 1835, Mr. Perry examined the limb with care. The purring was then not perceptible for more than an inch on either side of the sheath of the vessels, neither could it be *seen*, as before; but required for its discovery that the hand should be applied to the surface. The superficial veins of the leg had become remarkably enlarged.

"I may be permitted here to anticipate the subsequent details of the case, so far as to say that these changes were sufficiently explained on dissection by the obliteration which was found to have been brought about in the vein, probably by the long continued pressure, and which, by diminishing the bulk of the circulating contents of the vessel, rendered that remarkable *bruissement* less obvious than before. The obliteration of the femoral vein also accounted for the enlargement of the superficial veins." 38.

On the evening of the 9th of September, 1835, the patient applied to be re-admitted, on account of great and sudden enlargement of the tumor, which had taken place a few days before, and had been preceded by much pain and throbbing.

On his admission the tumor was found enlarged to three or four times its former size, and upon the point of bursting. Under these circumstances, the femoral artery was tied on the following day, in the usual situation. The artery was nearly the size of the abdominal aorta, and its coats so thin that it looked like a vein. Much difficulty being experienced in carrying the point of the needle round the artery, apparently from adhesion at its back part, and its extreme tenuity rendering it very hazardous to employ any degree of force, it was deemed advisable to enlarge the opening in the sheath, and pass the ligature about half an inch higher up, which was effected without any difficulty. So attenuated and fragile were the coats of the vessel, that they actually gave way under the slight pressure made by the fingers of a gentleman, who held it with the view of assisting in the passage of the needle under it, and blood issued in a minute jet from its anterior surface. This accident made it necessary to observe the greatest caution in tightening the ligature, as it seemed not improbable that if drawn with ordinary force, it would completely divide the artery. The noose was therefore only drawn to a sufficient degree to stop the pulsation in the sac and lower portion of the artery, and as the bleeding from the small laceration before described immediately ceased, it was deemed that enough had been accomplished to stop the circulation through the vessel, and the wound having been closed by straps of adhesive plaster, the patient was carried back to bed. The vein was not seen during the operation, being completely concealed by the enormous dilatation of the artery.

In the night of the 15th, there was profuse hæmorrhage from the wound, succeeded by syncope. Next day, the hæmorrhage recurred, and, on the sixth day after the operation, the patient died.

*Dissection.* Passing over less important particulars, we may note—the pericardium universally adherent to the heart—the right auricle large—the left ventricle hypertrophied and dilated.

Much atheromatous, and one spiculum of bony matter, in the coats of the aorta at its arch. This was much dilated at the origin of the arteria innominata, which was unusually large. The abdominal aorta was rather thin in its coats.

" The external iliac arteries, especially the left, were extremely tortuous, being reflected upon themselves in a singular manner, during their course towards the crural arch : a condition which had, no doubt, given rise to the impression of the artery being extremely dilated, since it conveyed to the hand simultaneously the combined pulsations of the folded portions.

The coats of the femoral artery, throughout its whole course, were scarcely, if at all, thicker than those of a vein, the attenuation having, as careful dissection afterwards proved, taken place equally in all its coats. Immediately below the origin of the profunda the vessel was greatly dilated, having the appearance of an aneurismal sac. Its coats were here softened and much attenuated, and presented an aperture anteriorly, large enough to admit the point of the ring-finger, from which the fatal hæmorrhage had taken place. The ligature had been placed at a very short distance below this part of the vessel. The wound was full of coagulum, which had also made its way into the sartorius and surrounding muscles, and by its pressure had probably restrained the hæmorrhage so as to prevent the patient from bleeding to death at its first access.

At the spot in the thigh where the communication had been presumed to exist between the artery and vein, there was an aneurismal sac, about as large as half a walnut, firmly ossified within, which, by the pressure it had exerted upon the vein, had caused absorption of its coats, so as to form a circular opening of about two lines in diameter, into whieh the aneurism had burst,—thus inducing a free and persistent communication between the vessels. Just below the aperture the vein was obliterated at a single *point,* below which it was again pervious. In all the rest of its course up the thigh it was diminished in size and thickened.

The changes above described, which took place in the limb between the time of the patient quitting the Infirmary and his re-admission, leave no room to doubt that the obliteration of the vein was accomplished in that interval, by the use of the spring, which effectually compressed the vessel under the bony walls of the aneurism.

At the lower part of the popliteal artery, exactly at the point of division into the anterior and posterior tibial trunks, the vessel was dilated into a very large aneurismal sac, which contained a small quantity of laminated fibrine ; but the greater part of its contents consisted of loose coagula and serum. The tibia formed a part of the walls of the sac, and was partly absorbed, so as to present a scabrous surface. The posterior tibial artery opened *from* the sac, as the popliteal had opened *into* it, by a circular and even orifice. The anterior tibial could not be examined, as it was sacrificed in separating the tumour from the tibia." 43.

There can be no doubt that the infrequency of such communications between an artery and vein, arises from the disposition of the latter to take on the adhesive inflammation, and to become obliterated by pressure.

2. *Remarks on two Forms of Atrophy of the Heart's Valves, which interfere with their Function ; founded on a Series of Cases. By Peter Nugent Kingston, M.D., Physician to the St. George's and St. James's Dispensary.*

Those who have the pleasure of Dr. Kingston's acquaintance are well aware that he is a zealous cultivator of pathological anatomy. They will receive his descriptions of morbid changes with confidence, as few take more pains to examine minutely the data for their pathological opinions.

A. *Anatomical Characters.*

*a.* " The lesion may be defined a simple shortening of the heart's mitral or tricuspid valve, without any diminution of its natural thinness, pliancy and transparency ; the orifice to which it belongs possessing at least the ordinary calibre. In other words, it is an atrophy of the valve in the direction of its length. The extent to which the valve is thus wasted is often considerable. Thus, the length of the posterior lamina of the mitral valve, which is naturally from seven to nine lines, was in one case reduced to three lines : in another it no where exceeded a line and a half, and was for the most part only a line. The laminæ of the tricuspid valve are naturally from eight to eleven lines in length : but in one of my cases they all three fell very short of this, and one was only three lines. The valves in question are generally thinner than is natural : but as attenuation does not interfere with the fulfilment of their function, it does not of itself constitute disease." 91.

*b.* Another lesion of the cardiac valves is nearly allied in nature and effects to the preceding. The continuity of a valve, so affected, says Dr. Kingston, is interrupted by apertures, sometimes of large size, and sometimes so numerous as to reduce the structure to a mere net-work, while the remainder is in a state of attenuation which is here and there often extreme, especially towards the edges of the aperture. Sometimes a large gap is seen, subdivided only by a few thready fibres. These, in the case of the auriculo-ventricular valves, are generally prolongations from chordæ tendineæ. In four of the cases which Dr. K. has seen within the last three years, the lesion had gone to a sufficient extent to have materially interfered with the valve's function. Laennec and Dr. Corrigan have both alluded to this affection.

*c.* It now and then happens that valves which have become thickened and indurated in various ways are shortened or cribriform. In a case cited by Burns, on the authority of a friend, it is said, "The mitral valve was indurated and reticulated." In a case which occurred to Dr. Kingston, (that of a waiter aged 27, who had had several attacks of acute articular rheumatism,) the posterior lamina of the mitral valve was yellow, opaque, much shortened, and the sixth of an inch in thickness. The adjacent portion of the inner membrane of the left auricle, for the extent of about two inches, was much thickened and corrugated, opaque, and of nearly the same hue as the valve. The whole heart was dilated and hypertrophous: the left auricle had twice the capacity of the right.

### B. *Comparative frequency.*

"That these are far from being rare lesions in comparison with the others to which the heart's valves are subject, may be inferred from the circumstance that out of about thirty fatal cases of diseased valves of which I took notes during a definite period, one or other of these species of atrophy existed in ten. The mitral valve was shortened in five, the tricuspid in five, both in two. In one the mitral valve was cribriform, in two the tricuspid, and in one both the aortic and pulmonary valves were so." 94.

Dr. Kingston accounts, in a satisfactory manner for the lesions being overlooked. They are such as would not strike the eye unless the valves were very minutely examined.

### C. *Functional Effects and Diagnostic Signs.*

A valve which has become shortened or cribriform, is, of course, incompetent to close completely the orifice to which it belongs, and consequently permits regurgitation. Such regurgitation must be in the ratio of the deficiency of the valve.

The effects of such regurgitation need scarcely be insisted on, as they must correspond with those which result from regurgitation from other causes.

"In all the cases in which the circumstances are fully detailed, there had been anasarca and palpitations; and in all but one, dilatation of some cavity posterior to the affected valve. In all in which the valves of the left side were defective, there had been dyspnœa and cough, pulmonary congestion and inflammations; the radial pulse and the præcordial impulse were frequently irregular and unequal; and out of the only four of these cases in which the history is sufficiently known, one patient was very subject to attacks of faintness and debility, another to vertigo and epileptic fits, a third died in a fit of syncope, and the fourth, after total sleeplessness for a fortnight, sunk into a state of coma which was fatal in a few hours. Where the defect lay in the mitral valve, the pulse at the wrist, compared with the beat at the heart, was generally small and weak; and where in the tricuspid, there was distention, sometimes attended with pulsation of the external jugular veins; in those cases, at least, in which they were examined. In several, the symptoms could not have been fully accounted for, had these valvular lesions been overlooked." 98.

Dr. Kingston is satisfied, from his stethoscopic examinations, that a bellows sound is often produced by the reflux of blood through the affected valve.

D. *Mode of Production and Causes.*

Dr. Kingston contends, and no doubt with justice, that these affections of the valves most commonly occur at a more or less considerable period after birth. The apertures in the valves he attributes to progressive absorption—the diminution of length to a similar process.

The remote causes of the shortening of the mitral and tricuspid valves are considered by Dr. Kingston to be essentially two—pressure, and a deficiency of nutritive action or power in the valve.

"In nine out of the ten instances in which the mitral or tricuspid valve was shortened, there was hypertrophy of the corresponding ventricle, which is in general preceded as well as attended by an increased force of the heart's contraction. In several, there was likewise obstruction to the discharge of the blood from the hypertrophous ventricle. In most, the patients had followed laborious occupations; and two traced their complaints in a great measure to over-exertion.

In these nine instances, therefore, the blood's impulse against the valve affected had been permanently increased, and the valve had hence been subjected to an unnatural degree of pressure, an agent very frequently observed to occasion the atrophy of almost every tissue and organ in the body." 131.

The causes of the cribriform atrophy next occupy our author.

"Of this there were five cases, in one of which the pulmonary and aortic valves were both affected: in two the auriculo-ventricular valve was shortened as well as cribriform.

One of these patients had been very subject to gout and to precordial palpitations; and two had had several attacks of acute articular rheumatism, attended with well-marked symptoms of inflammation of the heart; and after death exhibited old adhesions of the pericardium, and disease of some of the valves similar to that which is known to be consequent on rheumatic affection of the valves." 107.

Dr. Kingston hypothetically asks if gout or rheumatism could so impair the nutritive powers of a part, as to cause it to be absorbed under ordinary pressure? This question he anologically answers in the affirmative.

In the remaining two of the five cases, the cribriform atrophy was referable to the causes which have been assigned for the shortening; namely, to an undue force of the blood's impulse, combined with debility of the valves' nutritive powers from previous disease. The first circumstance was proved in both by hypertrophy of the corresponding ventricle, and obstruction to the discharge of its blood: the second was in both rendered probable by marks of chronic disease of the heart's valves in other places.

E. *Prevention and Treatment and other Practical Bearings.*

On these points it must be owned that there is not much to be said, and that what there is must be obvious from general considerations.

The great point is to avoid the general causes of cardiac disease—anxiety of mind, inordinate exertion of body, the metastasis of gout and rheumatism. The next point is to detect the lesion *if* we can. The third point is, to treat the case according to the symptoms, but with a view to these affections. The general principles on which diseases of the heart are to be managed, do not require especial notice here.

The paper of Dr. Kingston is one of interest to the pathologist. The mere practitioner will not see in it much to arrest his attention. But such papers are of value to science.

### IV. Affections of the Lungs, &c.

### 1. *Case of Diaphragmatic Hernia. By William Norris, M.D. of Stourbridge.**

Thomas Smith, aged 19, has, from childhood, complained of pain in the left side of the chest, and sometimes of slight cough, and difficulty of breathing; has looked sallow and thin, with the appetite rather deficient, and generally has had an excess of thirst; and for the last two years he has not been able to lie on the right side; but his sufferings have been so slight, that they never precluded him from following his occupations for a single day. Nearly two years ago he had a wrestling fight; after which, the pain increased for a short time, attended with sickness; but these symptoms disappeared in a short time. For nearly three years he has been a gentleman's servant. About seven weeks before his death he walked eighteen miles on one evening with ease.

It was last Summer when Dr. Norris was requested to visit him. He complained of pain over the whole abdomen, particularly in the left hypochondriac region, and in this situation the pain was much increased on pressure: there was considerable distention; and frequently vomiting of a porraceous matter, similar to the secretion from the stomach of a patient labouring under painters' colic; with much turning and tossing in the bed. Tongue covered with a whitish brown fur; no alvine evacuation for four days, excepting a few scybala; pulse 130, and soft; thirst excessive; countenance full of anxiety and suffering; respiration rather hurried, but not laborious. The bowels were often very torpid: early that morning he had taken a dose of salts, which had operated freely. Could assign no cause for his complaints: he had been gathering moss that same morning, and had climbed up a very steep hill, carrying a load of the moss upon his shoulders, and in the evening he was suddenly attacked with pain. He had been twice bled; blood not buffed, nor cupped; and had taken a variety of cathartics.

It was on the fourth day of the attack that Dr. Norris saw him, Mr. Granes, a surgeon, of Clent, having treated the case previously. Dr. N. looked on the case as one of mechanical obstruction, and suspected that it might be diaphragmatic hernia. Powerful cathartics, the cold dash, bleeding, were all tried, but unsuccessfully. The symptoms grew worse and worse, and on the next morning at eight o'clock the patient died.

---

* Provincial Transactions.

*Dissection.* On opening the abdomen, which was greatly distended, the omentum was not to be seen; all the bowels were filled with flatus, and in different stages of inflammation, varying from a tinted redness to a lurid hue; and small patches of sphacelus were commencing on the large intestines. On pushing the fingers downwards, the descending arch of the colon, and the pancreas were not to be found in the abdominal cavity; and on opening the thorax there was discovered a large knuckle of intestine, eight or ten inches in length, in the left cavity, which had dragged with it the greatest part of the omentum, and the whole of the pancreas. The lung had lost its natural structure, and more resembled spleen; and was condensed into the size of a man's fist. An aperture was discovered in the left portion of the diaphragm, near the vertebra, more than an inch in diameter. The bowel had contracted firm adhesions to the neck of this aperture. The omentum and pancreas were much thickened. The strangulated portion of bowel was of a mulberry tint, resembling bowel in the last stage of strangulated hernia, but the coats were much indurated, evidently showing the prolapsus to be of no recent date. There was more than a quart of very turbid fluid in the left cavity of the chest. The descending colon was much contracted and empty. The right lung appeared quite healthy. The pulsation of the heart had been felt to the right side of the chest; the heart having been forced from its natural situation to the upper part of the left cavity of the thorax.

Cases of diaphragmatic hernia, though not very uncommon, are sufficiently so, to render any which occur worthy of being placed on permanent record.

2. *Dissection of a Case in which one Lung had formerly been much compressed by fluid effusion.*

In the Provincial Transactions, there is a paper by Mr. Windsor, surgeon to the Manchester Eye Institution, on the Effects of Chronic Pleuritis, from which we extract the following notes of a dissection. We extract them because they shew the actual condition of the parts, long after a pleuritic effusion has been absorbed.

An eminent artist, of Manchester died at about the age of 28, and the examination of his body took place on the 14th of January, 1832. He had always been an invalid, at least from the time of having the measles, when very young; and had suffered much from pectoral complaints, as coughs, dyspnœa, &c.; his heart beat on the right side; he had latterly, also, been œdematous in the lower extremities. He was of short stature, rather emaciated, and a little deformed; the dorsal vertebræ inclining a little to the right side.

*Dissection.* There was scarcely any effusion in either pleura, but considerable adhesions on both sides. On the left side a light spongy cellular texture intervened between the ribs, and the pleura covering the lung and the pericardium; it was of considerable thickness and occupying a considerable space. The lung itself was void of crepitus, of small extent, being compressed against the posterior part of the thorax, and altogether carnified. The pericardium contained a little bloody serum, perhaps an ounce. The heart appeared of natural size; its right side, especially the right auricle, the cava superior and subclavian veins were distended with blood; the internal structure of the heart was pretty natural, the base of it was situated on the right side, the apex pointing sinistrad, reached just the mesial line. The internal membrane of the respiratory canal throughout was considerably injected.

No doubt, there had formerly been considerable fluid effusion in the left pleural cavity—the fluid had been gradually absorbed—the lymph had been converted into cellular tissue, the lung first compressed, had then, from that compression become carnified. The other lung only being employed in respiration, explained the dyspnœa. The immediate cause of death would seem to have been bronchitis.

3. *Researches on Some Points of the Pathology of Pulmonary Tubercles. By Peter Nugent Kingston, M. D., &c.*

Dr. Kingston observes, with truth, that it is an object of no small importance to perfect our acquaintance with the pathology of pulmonary tubercle. He endeavours, in this paper to shew:—1st, that the common pulmonary tubercle, is a vascular texture; 2dly, that it sometimes originates in an alteration of the air-cells and their secretions; and 3dly, that now and then it is entirely healed, when it has even extended over a very large portion of the lungs.

A. Tubercles have been concluded to be unorganized because they have not been, and could not be, injected. Dr. Kingston adduces many arguments to show that this is no proof of absolute want of organization. This may be granted, and if there are any circumstances which tend to establish the existence of organization, the incapability of receiving injections would certainly not invalidate the inferences that might be drawn from them.

The proofs of the organization of tubercle adduced by Dr. Kingston are the following:—

"Having shown the invalidity of the evidence adduced to prove the non-vascularity of pulmonary tubercles, I proceed to give an account of seven cases in which great numbers of pulmonary tubercles of the ordinary kind were distinctly seen to be traversed by red vessels.

They had not in their symptoms differed from the generality of cases in which tubercles have been found in the lungs after death. Cough, dyspnœa, pain of chest, night-sweating, diarrhœa, emaciation, had most of them been present in all. The subjects were of all ages, 7, 15, 21, 26, 42, 61, 65. In each, the lungs presented every stage of tuberculous disease. In some places there were transparent or semi-transparent, colourless or whitish bodies, many of them rounded or oblong, varying in size from that of a grain of sand to that of a hemp-seed, from cartilaginous to fleshy firmness. In other places, the bodies were nearly or altogether opaque, and of a whiter or yellowish hue; some were firm, others softened or quite liquid. Excavations had been formed, principally at the apices of the superior lobes. In two of the cases the tuberculation had for the most part assumed the form which has been termed infiltration.

In every one of these cases a great number of the isolated solid tubercles, both such as were white or yellowish and opaque, and such as were greyish and translucent, presented the following appearances. A careful section being made of the tubercle intended for examination, the cut surface was seen by a strong magnifying glass, sometimes also with the naked eye, to be traversed by continuous red lines, which were sometimes short and unconnected, but often of considerable length and making frequent ramifications and anastomoses, quite after the manner of small bloodvessels. An intelligent friend, to whom I shewed the appearance, compared it to the arrangement of vessels in an inflamed conjunctiva. On several trials we found that these red lines could not be removed by being gently scraped with the edge of the scalpel. In many instances they might be traced from the centre to the circumference of the tubercles, whence they were sometimes seen to extend into the adjacent pulmonary parenchyma, or to communicate with the vessels in the neighbourhood. There is no room for doubt that these red lines were bloodvessels: for such an appearance, when exhibited by solid parts, has always been considered decisively characteristic of vascularity.

In each case the number of tubercles in which red lines were visible was very great. When the portion of lung had grown stale by keeping, then, along with the red lines, there were some of a brownish red colour, and others which were dark-brown, or quite black. In all there were many tubercles in which no vascular appearance whatever was discernible. But it frequently happened that the pulmonary parenchyma, for a considerable distance around, was equally destitute of the appearance of red vessels. Indeed it may be observed of every tissue and organ in the body, that after death its vessels are sometimes filled with red blood, at other times empty, and that some parts are reddened, while others remain pale.

Whether those tubercles in which no vessels were to be seen after death were furnished with red blood during life, may therefore be questioned. But nutrient vessels of some kind must be conceded to them. For how can it be supposed that, agreeing in all other physical characters with those in which red vessels were visible, they differed so fundamentally as to be destitute of organization? The same may be said of several cases of pulmonary tubercles, in none of which I could find vessels containing red blood." 311.

This must be admitted to be fair evidence. It is corroborated by the written statements of Dr. A. J. Sutherland, to whom Dr. Kingston shewed several of the tubercles, and who expresses his entire belief in their vascularity. It is difficult to suppose that all this was accidental, or that Nature should do in one case what she is not in the habit of doing in others. And as the organizability of tubercles tallies better with the changes which occur in them, than does the supposition that they are not organized, we may suppose that the facts described by Dr. Kingston are not illusions or exceptions to the common order of things.

B. The site of tubercles has been a matter of discussion. While some have placed them in the intervesicular cellular structure of the lung, others, and those the majority of the best pathologists, have either contended or have been inclined to believe that their real seat is the air-cells.

Dr. Kingston not only adheres to the latter opinion, but offers the following hypothesis of the mode of origin of, at least, one species of tubercle.

"In October, 1834, I witnessed the inspection of a subject whose lungs presented the following appearances. In the upper lobe of the right lung were several tough, grey capsules. Some of these were filled with matter which, at the circumference, was very tough, black, and opaque; but at the centre, partly of a black, partly of a dirty-white colour, opaque, of a caseous softness, in some places minutely granular, held together by cellular substance. Other capsules were filled altogether with the soft matter last described. At the roots of the lungs were some similar masses, and also several capsules enclosing hard earthy nodules. One of the latter, however, had perforated both its cyst and the parietes of a large adjacent bronchus, and was projecting naked into its cavity. Very thickly disseminated through both lungs, were bodies considered by all present to be incipient tubercles. They were in general of a somewhat rounded or oval form, from the size of a grain of sand to that of a mustard-seed, some transparent and colourless, others whitish and semi-opaque; they felt hard and quite distinct from the adjacent pulmonary structure, as the finger was passed over them. On further examination, I found that each of these bodies consisted of a tough, nearly transparent cyst, filled with fluid, which in some was white, semi-opaque, thick and viscid, in others was thinner, transparent, and colourless. The neighbouring bronchi were filled with mucus of similar appearance, and in several instances a minute bronchus so filled could, on dissection, be distinctly seen to terminate in one of these vesicles. The bronchial mucous membrane in these parts was thickened; in some places white and semi-opaque, in others reddened and softened.

There could be no doubt that the tuberculoid bodies were continuous with the bronchi, were portions of the air-passages: and thence I learnt that the terminations of the air-passages, the air-cells as they are termed, are liable to become thickened, dilated, and filled with thick mucus, and that they then assume an appearance nearly resembling that of tubercles both to the eye and to the touch." 320.

Supposing the cavities of these thickened vesicles obliterated, either by solidification of their contents, or by thickening of their parietes, they would appear or be identical with miliary tubercles. Dr. Kingston offers several reasons for concluding that such changes are consistent with what we know to take place in the body, and he clenches the argument of analogy with one of fact. He states that, in two cases, the chain of proof was complete, and all the following particulars were presented.

1. Excavations, principally at the apices of the lobes, of the ordinary appearance of those which accompany tubercles.

2. White or yellowish, opaque or nearly opaque bodies, appearing in the section of the lung to have a somewhat rounded or oblong form, some nearly liquid, others of various degrees of firmness up to that of the pancreas.

3. Firm bodies differing from the latter only in having less colour and more translucency: many of those which at the circumference were very translucent and nearly colourless, having in the centre a small, yellow, opaque speck: others having a whiteness and tendency to opacity, slight towards the circumference, gradually increasing towards the centre. Some of these bodies, upon a minute examination, were found to be cylinders with a bulbous extremity, containing in some instances a yellow, opaque particle.

4. Bodies which did not to the eye differ from the preceding, some being nearly colourless and very translucent throughout, others of a whiteness and opacity gradually increasing towards the centre, the greater number of a somewhat rounded or oblong form: feeling hard also as the finger was passed over them while they lay in the lung. Upon a careful examination it was found that these consisted of membranes of various thickness, of a white or greyish colour, filled with colourless or whitish, translucent, very viscid fluid, having all the appearance of mucus: and in numerous instances it was observed that the adjacent bronchi were filled with mucus of the same appearance. Minute bronchi distended with this mucus could often be seen proceeding for a considerable distance, and, at last terminating in one of the vesicles filled with a similar fluid. There could, therefore, be no doubt that the vesicles were portions of bronchi, or of the cells in which they terminate, greatly distended with mucus. Particles of yellow, opaque, friable matter were, in some instances, contained in the fluid which exuded from the vesicles. Some of these bodies, which at first had the appearance of being single vesicles, were, on a closer scrutiny, discovered to be clusters of smaller ones.

5. Bodies forming a transition between the third and fourth groups: in which the central cavity filled with mucus still remained, but had been greatly diminished by the concentric thickening of the parietes. These bodies, likewise, were in several instances much elongated, having all the appearance of minute bronchi thickened.

He does not therefore doubt that the vesicles and solid tubercles were different stages of the same disease, nor that they equally consisted in an alteration of the minute air-passages, which at first became somewhat thickened and distended with their secretions, and were at last totally solidified, partly by the concentric thickening of their parietes and partly by inspissation of their contents.

c. The possibility of the cure of consumption, after ulceration has taken place, has been a subject of anxious speculation. The best pathologists and most experienced physicians admit that, when tubercles are limited to a small part of the lung, cicatrization of a vomica may now and then occur. But, in the following case, the disorganization had gone to an extent which, in the opinion of most men, would render recovery impossible.

*Case.* A coachman, aged 49, was admitted into St. George's Hospital in August, 1834, for a wound on the calf from the kick of a horse. He was cured in four months. He made no complaint of his chest to the surgeon, but the following particulars were ascertained from the nurse. He had an occasional severe fit of coughing, with some expectoration in foggy weather, but was often not heard to cough for many days together: never complained of pain at the chest, palpitations or dyspnœa, but was observed, while speaking, to take breath rather oftener than was natural: never required to be propped up in bed, lay generally on the left side, though sometimes on the right and sometimes on the back: was angry when once it was hinted that he was asthmatic. He did not sweat at night: required rather more than the usual allowance of bedclothes to keep him warm. He eat the whole of the "ordinary diet" with apparent appetite: used to be dressed and sitting up the greater part of the day, and for some time before he left the hospital could walk three or four miles without fatigue or any ill effect.

In the beginning of January he was discharged cured. A few days afterwards he was drenched by a shower, and was taken back to the hospital with symptoms of inflammation of the kidneys. Headache, delirium, and symptoms of effusion on the brain supervened, and on the twelfth day of this last attack, he died. He never made the least complaint of his chest.

*Dissection.* There was a good quantity of fat. The lower extremities were rather œdematous. Much serous effusion on the brain. Inflammation of the kidneys and bladder, the prostate gland containing pus. A few small ulcerations in the colon.

*Lungs.* "The left lung was loosely connected to the ribs and pericardium by a tough net-work containing a layer of fat from half to three quarters of an inch in thickness, and many inches in extent. The lung, when separated from this, was not larger than a moderate orange flattened; it had the breadth of the palm of the hand. The whole of the upper and part of the lower lobe was covered with a thick, reddish ligamentous membrane, from a line to a line and a half in thickness.

The lung had totally lost its natural sponginess, and was irregularly hardened. The upper lobe was entirely converted into a large excavation, with the exception of a rind which was in general from half a line to a line and a half in thickness, and no where exceeded three lines. This excavation was capable of holding above a quarter of a pint of fluid: it was slightly divided into three compartments by intersecting bands of pulmonary substance. In the lower lobe were two cavities of the size of small plums, and there were about a dozen as large as unshelled filberts, communicating with the former, some by large, others by small apertures. They were in some places within a line of the pleura, but in no place were they in contact with it. There was about twice as much of the lower lobe remaining as of the upper: they were separated from each other by a tough, somewhat vascular, fibro-cartilaginous tissue, which in some places was nearly half an inch thick.

All these excavations and the bands which traversed them were lined with membrane which was smooth, thicker than the mucous membrane of the bronchi, semi-transparent, generally whitish, but in some instances reddish, having the firmness in some places of mucous, in others of fibrous membrane. Here and there were whitish or black elevated points, generally covered by the inner membrane. Underneath there was generally a second membrane, which was whitish, fibro-cartilaginous, of the thickness of writing paper: but in a few places it was not very distinct from the tissue external to it. I observed the mouths of several large bronchi, some of the diameter of writing quills, opening into the excavations nearly at right angles with their surface, so as to have the appearance of being cut short off. Their mucous membrane was gradually lost at the edge of the excavation. At the mouth of one of the bronchi was a relic of one of the corroded cartilages, making a pointed projection into the cavity of the excavation.

One or two of the minor excavations were partially occupied by solid, but friable, granular, nearly opaque masses, which did not adhere to the surface of the excavations, and were evidently the residue of old tuberculous secretion, of which the fluid particles had been absorbed. The other excavations contained nothing but a slight mucous exudation; no puriform or tuberculous matter whatever.

What remained of the pulmonary structure of this lung was totally destitute of air, very tough, flabby, of grey or reddish brown colour. Each lobe contained six or eight tough, whitish, translucent capsules, of the size of pins' heads, filled with opaque, yellowish, minutely granular, dry matter, held together by cellular threads. There was also a small ramifying tube filled with the same matter. The bronchi and blood-vessels were generally much shrunk. The bronchi were lined with a reddish mucus, and their inner membrane was slightly reddened, and softened. A portion of one was dilated to four times the natural calibre: its lining membrane was similar to the rest, except that it was more reddened, and here and there gathered up into transverse ridges, very characteristic of bronchial dilatations.

At the right side of the chest the pleuræ were in a few places adherent by tough membrane, which, at the edge of the lower lobe, contained a mass of fat. The condition of the right lung differed widely from that of the left. It was larger than is usual. It contained no excavations whatever. About three fourths were perfectly healthy, except that a good many of the air-cells were enlarged. The remaining fourth was taken up with solid masses, disseminated in considerable numbers through the upper and middle lobes, very sparingly through the lower one. Their size varied from that of a mustard-seed, to that of a filbert. There was one whitish, semi-transparent, firm tubercle of the size of a mustard-seed, which appeared to be contained in the thickened dilated extremity of a bronchus. A great many consisted of capsules filled with matter of different appearances. Their contents were in a few instances a congeries of granules, of the size of millet-seeds, of caseous consistence, generally yellow and opaque, but in one or two instances translucent and whitish, here and there appearing to be connected by a cellular web. In several instances their contents consisted partly of these yellow granules, and partly of harder black matter. Then there were five or six pea-sized capsules, filled entirely with black matter, of considerably more than caseous hardness. But the lung was still more thickly strewed with roundish black masses, some of woody hardness, without any investing membrane. One cyst contained a white, hard calculus. The pulmonary tissue around these masses was crepitating, and in all respects healthy. Some of the bronchi of this lung contained a whitish or reddish viscid mucus: and the cartilages at the divisions of some of the small bronchi were much thickened." 332.

This must be regarded as a very interesting and valuable case. It demonstrates, so satisfactorily that it is not necessary to dwell on its distinctive features, the cure of extensive tuberculation, and such a demonstration must greatly assist the practical physician in his attempts to succour those who are labouring under phthisis pulmonalis.

In conclusion, we agree cordially with Dr. Kingston in this sentiment:—that it is not unlikely that the proportion of cases in which pulmonary tubercles are actually cured, is many times greater than the proportion in which the cure has been demonstrated. We hope that Dr. Kingston will continue to labour with the assiduity he has now evinced in the field of pathological anatomy.

4. *Observations on the Diagnosis of Pneumonia. By Dr. Addison.**

The main object of Dr. Addison's paper, and its modesty and utility combine to render apology needless, is to make some addition, however trifling, to the ordinary means of diagnosis of pneumonia—a complaint too frequently and too fatally overlooked.

A. The first point on which Dr. Addison offers a remark of any novelty or consequence, is the mode of origin of tubercles and of tubercular infiltration.

He observes that when, in cases of pneumonia, the albuminous matter thrown out in the air-cells is of a more than usually plastic or organizable kind, it is not entirely absorbed, but a portion of it permanently remains. At an after-period, it is found in small, detached, and more or less rounded masses, and receives the term of tubercles—a term which Dr. Addison thinks far too indiscriminately applied;—or, it is more extensively and irregularly diffused, when it is regarded as a form of tubercular infiltration.

There can be little doubt, that Dr. Addison is right in these sentiments. He does uot mean, of course, that tubercles have commonly this origin, or that tubercular infiltration is always to be referred to this source. But the

* Guy's Hospital Reports.

experiments of Cruveilhier and of others have shewn decisively that ordinary inflammation will give rise to tubercles, or to what may be regarded as such. The object of Dr. Addison's remark, is evidently to recommend greater strictness in the application of the terms, tubercle and tubercular infiltration. It is well to recollect that in common use and parlance they represent different forms of deposition.

B. But the main intention of our author is to elucidate the diagnosis of pneumonia.

He refers, in the first instance to the symptoms enumerated by Laennec as characteristic of the disease. They are, an *obtuse and deep-seated pain in the chest, dyspnœa, hurried respiration, cough,* and *peculiar expectoration:* but, in reference to these, Laennec also tells us, that each of them individually may occasionally be absent, and, indeed, that they may all be absent in the same case.

Dr. Addison contends that these symptoms are by no means so universally characteristic of pneumonia, as Laennec's description would imply, and that the exceptions are extremely frequent, and occur at every period of life, and in every variety of constitution.

Dr. Addison believes that pneumonia, such as the symptoms alluded to above represent it, is really not the simple, but a complicated form, and that the belief in its being the simple, is daily leading to serious oversights and errors. The following extract cannot, in fairness to Dr. Addison, be curtailed.

" In *simple pneumonia,* after chilliness, shivering, feebleness, and depression, the patient experiences, for the most part, strongly-marked symptoms of febrile re-action, giddiness, confusion, and sometimes intense pain in the head; occasionally delirium, especially towards night; *the skin acquires a pungent heat,* generally accompanied by dryness, more rarely by moisture; the pulse is full and strong, perhaps labouring and sluggish; the face is usually more or less suffused with a livid flush, accompanied by an expression of distress; the tongue is foul; its substance is more injected than in ordinary phlegmasiæ, and in a short time it manifests a tendency to become dry and brownish; the respiration is somewhat hurried, but *there is seldom any very obvious cough or expectoration, and sometimes none at all;* in short the whole assemblage of symptoms bears a most striking resemblance to those of a severe attack of common continued fever of the typhoid type, for which it is so repeatedly mistaken. If this form of the disease occur in moderately good constitutions, and is overlooked, especially if stimulants be administered on the supposition of its being a severe case of typhoid fever, it very commonly happens that the general prostration increases, the delirium or oppression of the brain is aggravated, the tongue gets dry and black, and the teeth covered with sordes; the breathing becomes more hurried, occasionally with a frequent slight hacking cough, and now and then a little bloody expectoration; the pulse gets flaccid, frequent, and feeble; and at length the patient dies.

Notwithstanding its close resemblance to a severe attack of continued fever— a resemblance so great, that even the stethoscopist is occasionally thrown off his guard—attentive observation will, in most cases, enable us to recognise the difference. The attack, in general, is more abrupt, and often follows some manifest exposure to cold or wet. The countenance, though congested and somewhat distressed, has not the dejection and stupidity so remarkable in fever: it displays more intelligence; and, although confused and perhaps slightly delirious, the patient, on being roused, commonly evinces a clearness and vigour of intellect not found in fever. The condition of the tongue also furnishes a valuable diagnostic sign. We know that, at the onset of fever, the contrast between the vividly-injected tongue and its white or grey fur is very striking: it is, in general, much less so in pneumonia. In the latter, if I may be allowed the expression, it is more the tongue of a phlegmasia: the hurry of respiration in pneumonia, is often not more than we commonly perceive amid the general distress of fever; and I repeat, that neither cough nor expectoration is necessarily present in a very appreciable degree. But of all the symptoms of pneumonia, the most constant and conclusive, in a diagnostic point of view, is *a pungent heat of the surface*. By this symptom alone, the first stage of pneumonia may, in most instances, be readily recognised: by this symptom alone, I have repeatedly pronounced the existence of pneumonia, before asking a single question, or making the slightest stethoscopic examination of the chest. The presence of this symptom has scarcely ever yet deceived me, even in the most complicated forms of inflammation within the chest. I by no means contend that it is necessarily present at some period of every case, although I do not know to the contrary; but I feel justified in affirming, that when inflammation is confined to the chest, however varied may be the tissues involved in the inflammatory process, provided this symptom be present, pneumonia may be confidently pronounced to form a part, in nineteen cases out of twenty, and I believe in a much larger proportion.

A similar pungent heat of the surface is now and then observed in certain forms of renal dropsy; more frequently in continued fever, especially in children; and still more commonly in the eruptive fevers of the exanthemata and erysipelas: and, as such cases may supervene upon already existing disease within the chest, the fact ought to be carefully remembered, lest a most valuable diagnostic sign should rather mislead than assist us. It is in original inflammation within the chest that it proves so constant and conclusive a sign of pneumonia, but on every occasion, when present, it ought to lead to a most careful scrutiny, by means of the stethoscope." 64.

The more simple form of pneumonia not unfrequently simulates affection of the brain, that is, the cerebral symptoms are most prominent. Dr. A. has seen several instances of this kind. In infants and very young children, the case not unfrequently apes hydrocephalus. In one instance, where hepatization had occurred, the symptom which attracted most attention was convulsions.

When there is cough and expectoration, Dr. Addison strongly suspects that the bronchial tubes are accidentally implicated, and that the amount of those symptoms is in the ratio of that implication. He is disposed to think that, in simple pneumonia, they are either not inflamed at all, or inflamed in a very slight degree.

When the mucous membrane of the bronchial tubes is involved to any considerable extent, the pneumonia is universally allowed to be complicated with bronchitis. It does not, however necessarily follow, continues Dr. A., that when pneumonia is present, the mucus of the accompanying bronchitis shall be tinged brown: on the contrary, the discolouration is often, in such cases, altogether absent, its presence and degree depending upon the quantity of blood which happens to be effused; exactly in the same manner as the ordinary *viscid sputa* of pneumonia may be colourless, or may be of a gamboge yellow, light green, or of a rusty or red colour, according to the same accidental circumstance.

Pain of any sort is seldom complained of in simple pneumonia, unless it is combined with pleurisy. But when the bronchial complication is such as to occasion severe cough, there is frequently a burning or tearing pain, or rather soreness, experienced.

We have laid before our readers a pretty close abstract of this paper. We have not noticed the stethoscopic signs, because, on them, Dr. Addison says nothing new.

Those who are inclined to agree with Dr. Addison, or to dissent from him, will observe that he lays down two propositions:—1, *that*, what is called simple pneumonia is really the complicated form; 2, *that* the symptoms of simple pneumonia are such as he has stated them, and not such as they are commonly believed to be.

The first proposition is rather stated than proved. To establish it we should have dissections of the simple and the common or complicated pneumonia, and it should be shewn that in the former there is no inflammatory lesion of the mucous membrane, whilst, in the latter, there is. Supposing these facts to be established, which they can only be by dissections, it will be impossible to deny that common pneumonia is really a complicated disease. At present we can only argue on probabilities and on general principles. Most persons will probably admit, that in common pneumonia the bronchial mucous membrane *is* affected. The difficulty is, to conceive any extensive inflammation of the lung in which it is *not* implicated. It is still more difficult to conceive that this should be a common occurrence. It is certain too that cases not unfrequently occur, in which common pneumonia, nay violent pleuro-pneumonia, takes place without giving rise to the usual symptoms. In such cases, the absence of cough and of expectoration cannnot be attributed to the freedom from bronchial complication.

Whatever view we take of the preceding question, we must necessarily be interested in ascertaining the value of the diagnostic signs laid down by Dr. Addison. They represent, or are meant to represent, an entity, simple pneumonic inflammation, and it matters little in point of diagnosis, what *its* relation to other forms of pneumonia may be.

It will be observed that they are all quantitative, rather than positive symptoms. The attack is *more* abrupt than in fever—the countenance is not *so* dejected—the intellect is not *so* confused—the tongue is *more* the tongue of a phlegmasia—the surface is *more* pungently hot than in fever. We need not hint that such symptoms of quantity, of plus or minus, necessarily in some degree, are dubious and deceitful, because they imply the existence of a standard which can generally be recognized. A few well informed men may, from experience, establish such a standard in their minds, but the bulk, perhaps, of the profession will find it difficult to do so. Yet it must be recollected that Dr. Addison rather advances these symptoms, as reasons for suspicion than conviction, and that they are calculated to induce the stethoscopist to apply his stethoscope, and discover the *rale crepitant.* But if a man is not a stethoscopist, what is he to do? Buy a stethoscope and learn to use it as fast as he can. This is only one reason amongst many for every man doing *that.*

5. *On Black Expectoration, and the Deposition of Black Matter in the Lungs, particularly as occurring in Coal Mines, &c. By William Thomson, M.D., of Edinburgh.**

This is a very long paper, and it must be owned rather a tedious one. The author might have saved his readers some trouble, by analysing the communications and the cases which he details in full, and we suspect that, had he done so, he would have increased the numbers of those readers.

We shall follow Dr. Thomson through the main heads of his essay, and transfer to our pages the facts and the considerations which we consider most important.

"There are obviously three ways in one or other of which the presence, in the texture of any part of the animal frame, of a substance not belonging to its natural constitution may be accounted for. 1st, It may have been separated from the mass of circulating blood by a process of secretion. 2d, It may have been discharged from the bloodvessels either through preternatural ruptures of their coats, or through their natural pores, without having undergone those changes in which secretion consists. And, 3d, It may have been introduced into the body from without, either by natural or artificial passages. In each of these cases, it is obvious, the substance may either retain the properties it possessed at the time of its being deposited in the situation in which it is found; or have undergone some subsequent change of properties in consequence of its being acted on by the surrounding solids and fluids of the body.

The presence within the body of different kinds of morbid matters of a black colour, has been accounted for on each of these three suppositions—that is to say, these matters have been conceived to be, in some instances, products of secretion; in others, to consist of extravasated blood; and in others, again, of foreign substances introduced into the body from without.

The frequent occurrence of black discolouration of the sputa during life, and the detection of black matter in the lungs and bronchial glands after death, more frequently than in any other parts of the body of which such matter does not form a constituent element, has led to the suspicion that in many instances these appearances must depend on the introduction of foreign substances into the respiratory organs by inhalation, and are not referable to either secretion or extravasation." 232.

It is the object of the author of the paper to ascertain the varieties of black sputa and black discolouration of the pulmonary organs, and to ascertain also their sources whether internal or the contrary. He is desirous, in particular, to determine the liability to these affections on the part of those who are especially exposed to the inhalation of carbonaceous powders or gases, such as coal-miners, and moulders in iron-works.

He divides the paper into three parts. Of these the first only is now published. It comprises all the individual cases of these affections, occurring in the class of persons alluded to, which have yet come to his knowledge —and, in addition, some communications relative to them, which have been received from gentlemen who have enjoyed the opportunity of witnessing them on an extensive scale.

A. "The cases which we have collected may be referred to two classes, 1st, Those in which the lungs have actually been found infiltrated with black matter on examination after death; and, 2d, those in which from the attendant symptoms, and particularly from the occurrence of black expectoration, there has

* Med. Chir. Transactions.

been reason to believe that such an infiltration had taken place, though an opportunity was not afforded of verifying the suspicion by anatomical examination. The first of these classes may be again subdivided under two heads; 1st, those cases in which there occurred symptoms of pulmonary affection during life, and in which for a longer or shorter period of time, the expectoration was of a black colour; and, 2d, those in which no mark of pulmonary ailment, or at least no black expectoration had been observed during life, and consequently no suspicion of black infiltration having occurred, was entertained previous to dissection." 234.

*a.* Dr. Thomson is acquainted with ten examples of cases of the first description; viz., of those in which there occurred during life black sputa and other symptoms of pulmonary affection, and in which, after death, black infiltration of the lungs was discovered. We shall subjoin the account of the state of the lungs in one case, in order that it may serve as a sample of the rest.

Both lungs, and particularly the right, were found to adhere strongly to the pleura costalis. The pleura pulmonalis of both lungs was much thickened, in some places, especially in the right lung, exhibiting a fibro-cartilaginous appearance and consistence, and about one-fourth of an inch in thickness. The pleura costalis corresponding to this portion of the pleura pulmonalis was ossified, and had caused bony union of several of the ribs. When cut into, both lungs presented one uniform black carbonaceous colour, pervading every part of their substance. The right lung was much disorganized, and exhibited in its upper and middle lobes several large irregular cavities, communicating with one another, and traversed by numerous bands of pulmonary substance and vessels. These cavities contained a good deal of fluid, which, as well as the walls of the cavities, partook of the same black colour. A considerable portion of the pulmonary substance surrounding them was dense, hepatized and friable. The rest of the lung was also somewhat condensed and very œdematous. The serum, when expressed, was of the same black colour as the substance of the lung. The left lung did not appear to contain any cavities, but was condensed and loaded with black serum. Some minute hard points could be felt in various parts of both lungs, but they did not differ at all in colour from the surrounding substance, and no distinct tubercular deposition or infiltration could be detected in those portions of the lungs which were most hepatized, even with the aid of the microscope. The texture in these parts appeared quite uniform, and the minute hard points felt in other parts, rather conveyed the impression of their being merely the ends of small bronchial branches divided on making the section. The bronchial glands did not appear enlarged, but partook of the same black colour as the substance of the lungs.

*b.* The second class of cases are those in which the lungs have been found, on examination after death, to be infiltrated with black matter, although there had been no black sputa during life. Six cases of this kind have come to the knowledge of our author. We shall mention the particulars of one case only. It is sufficiently illustrative of the fact.

*Case.* The patient had received a compound fracture laying open the left knee-joint and fractnre of the clavicle, scapula, and some of the ribs of the same side, by a mass of coal falling on him. The leg was immediately amputated, and the patient was doing well till the fifth morning after the accident, when he was suddenly seized with a very acute pain on the left side of the chest, confined to a small spot in the situation of the fractured ribs, and accompanied by severe rigour, for which he was bled, &c.; but he sank rapidly and died four hours afterwards.

*Inspection of the Thorax.*—A few ounces of bloody serous fluid and some adherent lymph were found on each side of the chest. The pleura costalis was ruptured, the lung not torn. The pulmonary tissue was very black, and afforded the black colouring matter with great facility when cut down and pressed in water. It appeared, however, sound, and to have suffered no change of structure.

c. The third class of cases comprehends those in which black sputa have been observed during life, but in which there has not been an opportunity of examining the state of the lungs after death. And in connexion with these cases are several communications from gentlemen practising in different districts in Scotland.

We shall, as before, adduce one case only in illustration, and pass on.

*Case.* Dr. Marshall, of Cambuslang, in an essay referred to more than once by Dr. Thomson, mentions that some time previous to his having had an opportunity of ascertaining the nature of this disease by dissection, two cases came under his care, which he now regards as examples of melanotic phthisis. The first occurred in 1825; the patient had been a coal-heaver the greater part of his life. He had been labouring under chronic rheumatism for some years, and had occasional attacks of cough, difficulty of breathing, and palpitation. He attributed the commencement of his ailments to having worked for some time in a very impure atmosphere. In June, his debility had increased so much as to confine him totally to his house; and along with this there was great emaciation, with aggravation of the cough and dyspnœa, and his expectoration now assumed a dark inky colour, which became deepened as his disease advanced. In September, he was reduced almost to a skeleton, and the black expectoration had become very profuse. At this time he was removed to another part of the country, where he died shortly afterwards.

We may now leave the consideration of cases of the affection, and examine the reports alluded to, for the purpose of ascertaining the views of Reporters on the cause of the complaint.

1. Dr. Marshall, of Cambuslang makes, the following observations on the origin of the disorder.

" In the first place, the use of gunpowder is strictly prohibited in the pits here, excepting in getting through stone, a work not entrusted to the regular colliers. Second, one of the individuals had wrought at stone work in driving a level, but not for a great length of time previous to the obvious development of the affection; and third, this same individual, while employed as above mentioned, was in a pit charged with a most impure atmosphere. I may remark, in conclusion," adds Dr. Marshall, " that after weighing all the circumstances connected with the cases I have seen, I still regard the deposition of the coal itself in the lungs as the cause of the disease." 267.

2. Professor Schœnlein, of Zurich, informed our author's father that he had seen several cases of the black infiltration of the lungs occurring in miners, both at Wurzburg and at Zurich, and was satisfied that, in some instances at least, the disease had appeared in persons belonging to mines in which no gunpowder was used.

3. Dr. Dewar, of Dunfermline, who has had great opportunities of observation, expresses the following sentiments :—

"You will please to observe the distinction between colliers and stone-workers. The former are employed merely at the coal-wall, and use only picks and wedges at their work; while the stone-workers are occupied, in whole or in part, in removing the free-stone and other rubbish which separate the different layers of coal. In these latter operations, the aid of gunpowder is constantly required, and the workmen, from the very imperfect state of the ventilation, are frequently enveloped in dense smoke. The persons who are exposed to this noxious atmosphere are, so far as my experience goes, the victims of this most fatal species of disease in the lungs, viz., consumption with black spitting.

1. Four brothers of the name of Smith, died with black spitting; all of them were stone-workers. Four sisters, of the same family, who have worked regularly in the coal-pit, are all in good health.

2. Four men, of the name of Bowman, died with black spitting; all of them stone-workers. Three brothers and four sisters, of the same family, who have worked in the pit, but not at stone-work, are all well. One sister died of consumption, the wife of one of the Smiths, but she had no black spitting. She died before her husband.

3. Two men, of the name of Brown, died with black spitting; both stone-workers. Three sisters, who have worked in the pit, are well.

4. Two Wilsons, stone-workers, died with black spitting. Three sisters, workers in the pit, well.

5. Four Campbells, stone-workers, died with black spitting. Three sisters and four sons, workers in the pit, well.

6. Williamson, a stone-worker, died with black spitting. Two brothers and one sister, pit-workers, well.

7. Black, a stone-worker, died with black spitting. Three sons and four daughters, workers in the pit, well.

8. Three Simpsons, stone-workers, died with black spitting. One son, a stone-worker, now dying with it. Two other sons and four daughters, pit-workers, all well.

9. Three Duncans, brothers. James, a stone-worker, died of the disease: Archibald, a stone-worker, now labouring under a cough with black spitting; and John, a collier, an old broken-down man, with a cough and difficulty of breathing, but no black spitting." 171.

He adds that all his patients have been somewhat advanced in life—that the progress in all was slow—and that he did not attend one case at the colliery among those employed where no gunpowder was used.

4. Mr. Philp, surgeon, of Aberdour, furnishes a very circumstantial report. He believes that the disease is occasioned by inhaling, for a length of time, the impure air of the mines, this impurity being caused by a mixture of carbonic acid gas with the atmospheric air. The following are his principal reasons :—

"All the individuals whom I have seen suffering from this disease were males. In tracing their history I have always found that they have worked less or more at what it technically called 'stone-work.' The stone-workers form but a small proportion of the workmen employed in a coal-pit, not more than twenty out of

130 or 140. Among the greater number who work constantly at the coal-wall, that is in heaving the coal, I have not found any instances of the disease to occur. In working at stone-work, that is in sinking pits and driving mines of communication, the workmen are exposed in an eminent degree to the influence of the impure air; for besides working in a confined space, and in a cul-de-sac, where the ventilation is very imperfect, there is also a considerable exudation of the carbonic acid gas from the fresh-cut surfaces of the minerals. In this impure air they continue to work for many hours daily for some months, their operations being frequently carried on several yards in advance of where their lamps will burn. In working at the 'coal-wall,' on the other hand, the workmen are in large roomy spaces, where every attention is paid to maintain a free ventilation. Amongst this class of workmen I have only met with two cases of the disease, some having worked for forty or fifty years as colliers without suffering at all."

5. Mr. Steele, of Craighall, says:—

"I consider the pulmonary disease of coal-miners to be excited chiefly by two causes, viz., running mines in stone, and working in impure air. In running stone-mines the workmen use gunpowder; there is often little and sometimes no ventilation; and the air is loaded with stony particles, with gunpowder-smoke, lamp-smoke, and sometimes, though not always, with choke-damp. This kind of work is a fertile source of evil, and if persisted in, sooner or later produces incurable disease in those who are engaged in it." 284.

6. Mr. Stevenson, of Silmerton, writes:—

"I have to state that in this place I have never yet been able to trace it (the black expectoration) to colliers at all, unless they may have combined mining operations with their usual work at the coal-wall. Indeed, from the cases that have fallen under my own observation, I have come to the conclusion that no collier or individual engaged in working coal only, either by blasting it with gunpowder or hewing it out in the ordinary way, does ever present the least expectoration of this sort while labouring under pulmonary consumption; but that it is confined entirely to those employed in stone-mining, or carrying through a communication below ground from one seam of coal to another, in certain kinds of hard rock, especially when, from the position of the strata or otherwise, much use is made of the pick; and I am further led to believe that some species of rock are worse in this respect than others. Some of the workmen suppose, that the use of gunpowder contributes greatly to the formation of the disease, but from what I have been able to learn, without any proper foundation. For although the spittle may be black after inhaling for a length of time the smoke arising from the different explosions in close mines, yet the blackness generally disappears within a few days, or a week at most, in the same manner as when it is induced by lamp-smoke, leaving the lungs perfectly sound." 292.

Such is the main evidence, so far as it has gone. Dr. Thomson forbears pronouncing an opinion upon it at present, as he is still engaged in the prosecution of the enquiry. When the remainder of his Paper appears we shall resume the subject. In the mean time, we may urge those who have opportunities of investigation to collect all the facts and ascertain all the circumstances which those opportunities afford.

We must think of bringing this article to a conclusion. Before we do so, we may cast a glance behind; and survey at a bird's eye view the ground over which we have advanced.

The substance of what we have presented to our readers has been taken from the Medico-Chirurgical Transactions. These are incomparably superior to any other Transactions which have been published, of late years, in this country, or, perhaps, in any other. Without being transcendental, the papers are, for the most part, highly scientific, and the too abstracted character of *mere* science is happily dashed with a strong mixture of practical observations. Cases with long diurnal details are banished from the pages of these Transactions, and though striking insulated facts are not excluded, they *must* be striking to obtain insertion. The policy of this procedure is equal to its dignity, for, as it is honourable to appear as a contributor, a supply of able contributions is ensured.

Our limits have not suffered us to notice *all* the Papers. The most important, indeed the great majority, have been extracted or analysed, and out of seventeen we have presented an account of twelve. Those which remain are:—Observations on Vascular Appearances of Mucous and Serous Membranes, as indicative of Inflammation, by Dr. Yellowly—On the Treatment of Injuries received in Dissection, by Mr. Stafford—Case of Unusual Dislocation of the Thigh-bone, by B. Travers, jun.—Sequel of the History of Mary Wren, by Mr. Birch—Removal of a Portion of Lung which protruded through a Wound, by Mr. Forde. These will be adverted to at sufficient length in our next number.

When we turn to the Guy's Hospital Reports, we see much which merits encomium. The spirit which led to the publication of the Reports is an honourable one, the mode in which that publication has commenced was a bold one, and the manner in which it has been carried on is creditable. The present number is rather superior than inferior to its predecessors. Of nine articles which it contains we have offered an analysis of five, and those the most important. The remaining four are:—An Essay on the Safety-Valve Function of the Right Ventricle of the Human Heart, by Mr. T. W. King —A Summary of Cases in the Obstetric Ward, by Dr. Ashwell—Description of a Remarkable Case of Urinary Calculus, by Dr. Hodgkin—Illustrations of the Museum, by the same.

The Transactions of the Provincial Medical and Surgical Association arrived late in the Quarter, yet have been noticed in this Number. Its contents are the following:—1, Some Reports of Anniversary Meetings, Councils, &c.; 2, the Report of a Committee on Medical Relief to the Sick Poor; 3, the Retrospective Address, by John Green Crosse; 4, Medico-Topographical Account of Bolton and its Neighbourhood, by James Black, M.D.; 5, On Glanders in the Human Subject, by James Johnstone, M.D.; 6, a Case of Ovarian Tumor, successfully removed, by W. Jeaffreson, Esq.; 7, on the Physiology of the Muscular Nerves of the Eye, by R. T. Hunt, Esq.; 8, on the Unity of Organic Structure, by Thomas Paris Dick, M.D.; 9, Cases of Encysted Dropsy of the Thyroid Gland, by Congreve Selwyn, M.D.; 10, on the Influence of Sleep on Digestion and Secretion, by R. W. Scott, M.D.; 11, Cases and Dissections, chiefly in reference to the Uncertainty of Diagnosis, by Thomas Poyser, Esq.; 12, Case of Tetanus, successfully treated by Carbonate of Iron, by J. Hamerton, Esq.; 13, Case of Loss of the Memory of Language, by T. Shapter, M.D.; 14, Case of Malignant Abdominal Tumor, by T. Salter, Esq.; 15, a Case of Diaphragmatic Hernia, by W. Norris, M.D.; 16, some Cases of Metastasis of Rheumatism to Internal Organs, by J. R. Walker, M.D.; 17, Facts Illustrative of the Effects of Chronic Pleuritis, by J. Windsor, Esq.; 18, Report from the Birmingham Eye Infirmary; 19, Report from the Birmingham Dispensary; 20, Report from the Worcester Infirmary; 21, Observations on Medical Relief to the Sick Paupers, by Messrs. N. Rumsey, R. Ceely, and H. W. Rumsey; 22, Observations on the Sick Poor, &c. by John Yellowly, M.D.

The second, the third, the fifteenth, the seventeenth, the eighteenth, the nineteenth, the twentieth, the twenty-first, and the twenty-second papers have been noticed at greater or less length in the present, or in the two preceding numbers of this Journal. What space we have now at our disposal shall be dedicated to some of the Papers which remain.

### Ovarian Tumor successfully removed. By Mr. *Jeaffreson*, of Framlingham.

In this case, the sac of an ovarian dropsy was partially exposed by an incision of ten or twelve lines in extent, its fluid evacuated by the trocar, and then it was excised from its attachments to the left side of the uterus. Mr. Jeaffreson was induced to perform this operation from the uniformly fatal result of all the cases of ovarian dropsy which he has had an opportunity of witnessing, He thinks that the indifferent success, which, on previous occasions, has attended the operation, has resulted from its having been delayed too long, in fact, until it has contracted too numerous and too firm adhesions to surrounding parts.

*Case.* In November, 1833, Mr. Jeaffreson was requested to attend Mrs. B. in her second labour. On making the usual examination, he discovered a tumor occupying the entire left half of the pelvis; it was firm, elastic, obscurely fluctuating, and evidently covered by the parietes of the vagina. At first it could not be got to recede, but by gentle pressure, it at last, suddenly passed above the brim of the pelvis, and the labour proceeded naturally.

On the 4th of March, 1836, Mr. Y. was again in attendance, and a fine boy was born without difficulty. But after delivery, the abdomen was little reduced in size, and very considerable effusion evidently occupied the left ovary. Various remedies were tried, with the view of procuring absorption of the tumor, but in vain.

On the 4th of May, she acceded to an operation proposed by Mr. Jeaffreson, the tumor being then on the increase.

"Accordingly, on the 8th, in the presence of my friend Mr. King, I made an incision of between ten and twelve lines, in the course of the linea alba, midway between the navel and the pubes, and having thus carefully exposed the sac, I evacuated by the trocar about twelve pints of clear serum. During the flow of the serum a portion of the sac was secured in the gripe of a forceps, to prevent its receding; and I afterwards gradually extracted the sac entire from the cavity of the abdomen, together with another sac containing two ounces of fluid, and, indeed, the entire ovary, having only to cut through a slight reflection of the peritoneum and ovarian ligament, which, with the exception of a small portion of the fimbriated extremity of the fallopian tube, are the only natural attachments of the ovary to the uterus. But as this part was the medium of vascular supply to the sac, and the vessels on the surface of the sac were unusually large, we thought it right to include it in a ligature previous to returning it into the cavity of the abdomen: the ends of the ligature were cut off close to the knot. A very small portion of omentum protruded with the sac, but was very readily returned; the external wound was closed with two sutures, adhesive plaster, and a compress of lint; and, by Mr. King's advice, I gave immediately a pill containing two grains of powdered opium, and a draught with a drachm of tincture of foxglove, keeping a napkin wrung out of the coldest spring water, constantly applied over the whole abdomen. This plan I the more readily adopted, as this gentleman considered the well-doing of a patient, into the cavity of whose abdomen he had occasion to make a much more extended incision, was very much promoted by it; an operation which, from its very important bearings, I sincerely hope he will be induced to give to the profession at no distant period. In the night I gave a dose of calomel and extract of henbane; and followed this by giving, every four hours, a solution of sulphate of magnesia in saline mixture." 243.

On the 10th, there came on excessive vomiting, hiccough, pulse scarcely to be felt, griping pain in the bowels, and an aching, shooting pain in the course of the anterior crural nerve. These symptoms were relieved by a stimulating enema, an opiate, and a slight stimulant.

The sutures were removed forty-eight hours after the operation, when the wound was healed every where except at the points of their application. A compress and plaister were re-applied, and the latter was put on, for the last time, on the 15th. It is not necessary to say more, as the case did perfectly well.

Mr. King, of Saxmundham, has repeated this operation on a lady, where the ovarian sac was much more distended, and having evacuated 27½ pints of fluid, he extracted it entire, together with a tubercular tumor the size of a turkey's egg. This lady has recovered without an unpleasant symptom.

These must be regarded as fortunate cases. The operation cannot be deemed other than a formidable one, under the most favourable circumstances. These cases, however, should be kept in the recollection of surgeons, and may encourage them to make attempts for the effectual relief of patients affected with ovarian dropsy.

### Case of Tetanus successfully treated by Subcarbonate of Iron. By *J. Hamerton*, Esq., of Elland.

Mr. T. N. aged 21, fractured the second phalanx of the thumb of the left hand, in the early part of February, 1835. Three weeks afterwards, there was much stiffness about the wrist, and general rigidity of the muscles of the body. On the 1st of March, Mr. Hamerton was called to him. He had then had some violent spasms, with opisthotonos, and was seized with one while Mr. H. was examining him.

The treatment consisted in bleeding to 16 ozs.—an electuary of the subcarbonate of iron and the spirits of turpentine—a turpentine enema—a blister to the pit of the stomach—friction with the camphor liniment—and subsequently tonics. By the 15th, the tetanic symptoms were gone. During the space of fourteen days, this patient took as much as a pound and half of carbonate of iron.

This cannot be looked on as a case of very acute tetanus, and the cure is therefore the less inconsistent with what we usually observe.

